# Reactive Oxygen Species Production and Mitochondrial Dysfunction in White Blood Cells Are Not Valid Biomarkers of Ageing in the Very Old

**DOI:** 10.1371/journal.pone.0091005

**Published:** 2014-03-10

**Authors:** Laura Wiley, Deepthi Ashok, Carmen Martin-Ruiz, Duncan C. S. Talbot, Joanna Collerton, Andrew Kingston, Karen Davies, Patrick F. Chinnery, Michael Catt, Carol Jagger, Thomas B. L. Kirkwood, Thomas von Zglinicki

**Affiliations:** 1 Institute for Ageing and Health, Newcastle University, Campus for Ageing and Vitality, Newcastle upon Tyne, United Kingdom; 2 Unilever Discover, Colworth Science Park, Sharnbrook, Bedfordshire, United Kingdom; 3 Wellcome Trust Centre for Mitochondrial Research, Institute of Genetic Medicine, Newcastle University, Newcastle upon Tyne, United Kingdom; University of Arkansas for Medical Sciences; College of Pharmacy, United States of America

## Abstract

Reliable and valid biomarkers of ageing (BoA) are needed to understand mechanisms, test interventions and predict the timing of adverse health events associated with ageing. Since increased reactive oxygen species (ROS) production and mitochondrial dysfunction are consequences of cellular senescence and may contribute causally to the ageing of organisms, we focused on these parameters as candidate BoA. Superoxide levels, mitochondrial mass and mitochondrial membrane potential in human peripheral blood mononuclear cells (PBMCs) and subpopulations (lymphocytes and monocytes) were measured in participants from the Newcastle 85+ study, a population-based study of the very old (aged 85 years and older). The intra- and inter-assay precision expressed as coefficient of variation (CV) for all parameters was acceptable (3% to 12% and 5 to 22% respectively). All parameters were stable in the short-term (1 week interval) in a sample of control individuals in the PBMCs and lymphocyte subpopulation, however they were unstable in the monocyte subpopulation; this rendered monocytes unreliable for further analysis. There was a significant association between superoxide levels and mitochondrial mass (positive in lymphocytes, p = 0.01) and between superoxide levels and mitochondrial membrane potential (negative in PBMCs, p = 0.01; positive in lymphocytes, p = 0.05). There were also significant associations between superoxide levels and mitochondrial parameters with other markers of oxidative stress-induced cellular senescence (p≤0.04), however some were in the opposite direction to expected. No associations were found between the measured parameters and age-related outcomes, including cognitive impairment, disability, co-morbidity and survival - questioning the validity of these parameters as candidate BoA in the very old.

## Introduction

The United Kingdom, like other high income countries, is undergoing dramatic changes in the age structure of its population due to increasing life expectancy and thus continuing growth in the older population [1]. Since the older population are more vulnerable to longstanding illnesses and disabilities and report the worst self-rated health, a major concern is an increase in the number of morbid years towards the end of life [2]. This highlights the importance of understanding the complex biology of ageing and its association with frailty and disease [3]. There are considerable differences between individuals with respect to the rate and extent of age-related decline, driven by a combination of genetic, stochastic and environmental factors [4]. Thus there is a need to find biological measurements that can discriminate between individuals who share the same “chronological age” but differ in their “biological age”. These so-called biomarkers of ageing (BoA) will be useful to understand mechanisms, test interventions and predict the timing of adverse health events associated with ageing [5]. Many candidate BoA have been proposed, including various anthropometric, physical, physiological, haematological and biochemical parameters. However, there are inconsistencies between studies and to date there are no measurements that meet the full criteria of a BoA [5]. Advances in the study of the biological mechanisms of ageing have identified various cellular and molecular markers, although there is little information on their role as BoA within the population, especially in older age groups.

Reactive oxygen species (ROS) are highly reactive molecules that contain an unpaired electron capable of taking an electron away from a target molecule in order to restore its stable state. ROS are important in many biological processes such as prostaglandin synthesis, immune defences, various enzymatic reactions and cell signalling processes. However, under certain circumstances, antioxidant defences become less efficient and ROS can cause structural damage to surrounding molecules including lipids, proteins and DNA. This results in the dysregulation of physiological functions increasing vulnerability to detrimental health outcomes [6].

**Figure 1:**
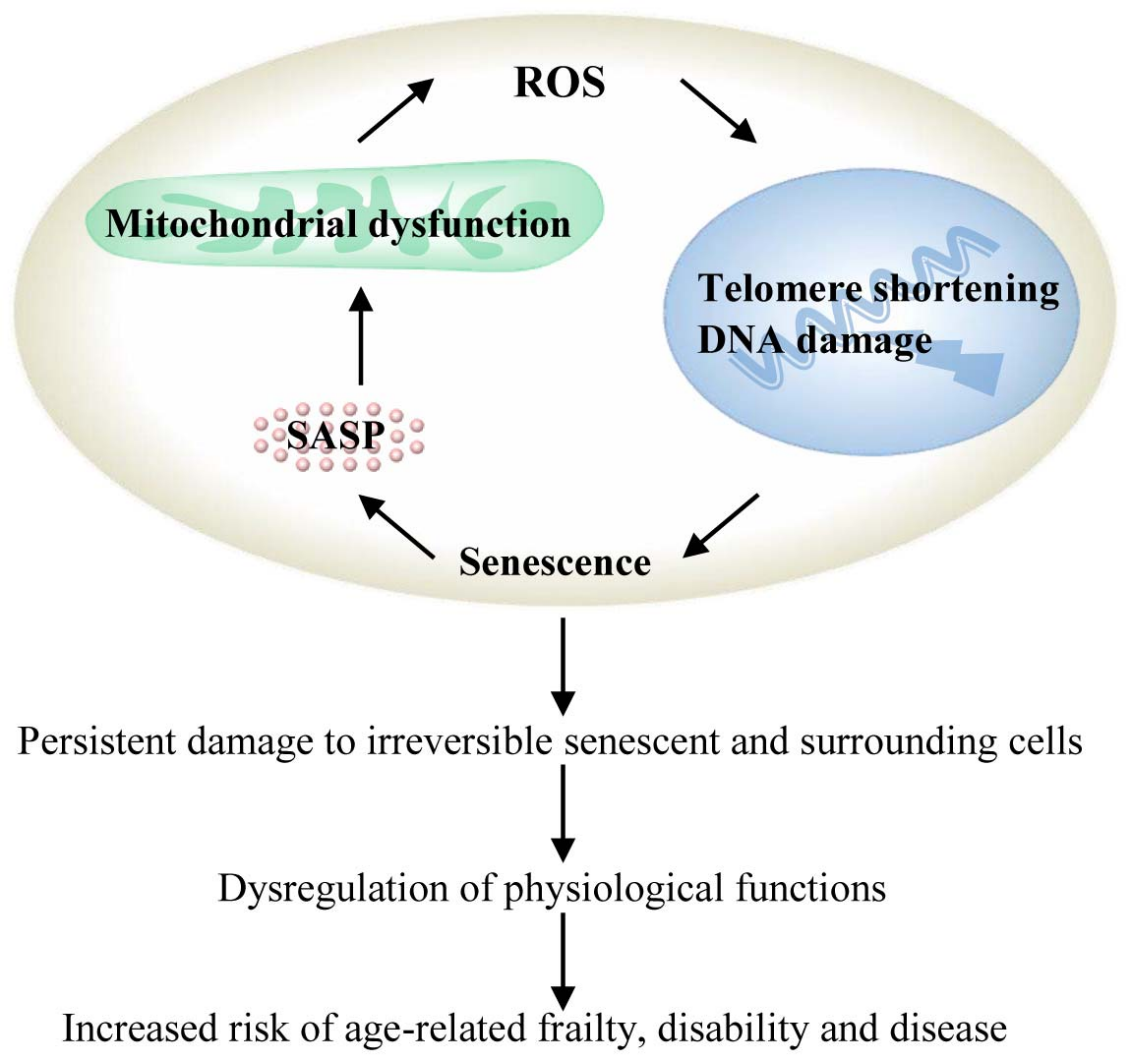
ROS production from dysfunctional mitochondria as the driving force of cellular ageing. ROS accelerates the onset of replicative senescence by increasing the rate of telomere shortening [42], [43] as well as feeding into a continuous DNA damage response that sustains the accumulation of inflammatory mediators as part of the senescence-associated secretory phenotype (SASP), and mitochondrial dysfunction, defined as enhanced production of superoxide together with (frequently) increased mitochondrial mass and decreased mitochondrial membrane potential. This maintains an irreversible state of persistent damage in senescent cells [22], [44]. The subsequent dysregulation of physiological function contributes to age-related pathology and/or carcinogenesis [45], [46].

Mitochondria are the major source of ROS within a cell. The main function of the mitochondria is the production of metabolic energy in the form of adenosine triphosphate. Although most of the oxygen consumed by the mitochondrial electron transport chain is reduced to water, a small proportion is converted to ROS, which may reach 1–2% in isolated mitochondria under specific experimental conditions [7]. Mitochondria themselves are a major target of the ROS they produce and are therefore subject to high levels of ROS-induced damage [8]. This in turn may induce further ROS production, when enzymes in the electron transport chain of the mitochondria become damaged directly or indirectly by ROS [9], [10]. Several studies have shown the relevance of increased ROS production from dysfunctional mitochondria as a major driving force in cellular ageing (Figure 1). ROS production and mitochondrial dysfunction are therefore potential BoA.

There are various steps to consider when investigating candidate BoA. Firstly, the degree of measurement reliability needs to be established. This is critically important in population studies to ensure the variability of the candidate BoA reflects genuine inter-individual differences and not measurement error. Therefore evidence of experimental and intra-individual stability should be shown. Experimental stability concerns various handling factors during preparation and analysis where significant effects can be controlled during experimentation. Intra-individual stability concerns factors of an individual that may impact on the day-day repeatability of the candidate BoA which can be controlled before experimentation or using statistical approaches during analysis [11]. Secondly, evidence of construct validity should be shown, which is the ability of a measurement to reflect known characteristics of the construct being investigated, in this case biological ageing [12]. BoA should therefore reflect known mechanisms of ageing and be associated with age-related outcomes [5]. Thirdly and most importantly, BoA should be able to predict the years of remaining good health and the trajectory towards a wide range of age-related outcomes and mortality better than chronological age [5], [13].

We aimed to assess 1) the reliability of superoxide levels, mitochondrial mass and mitochondrial membrane potential measurements in peripheral blood mononuclear cells (PBMCs) by flow cytometry and 2) their validity as potential BoA in a population-based longitudinal study of a birth cohort of the very old population (aged 85 years and older). Despite establishing methodological reliability, we did not find any predictive power in terms of longevity or age-related health outcomes in this age group.

## Materials and Methods

### Participants

This study was nested in the Newcastle 85+ study, a population-based cohort study of health and ageing in the very old, the methodology for which has been reported [14]–[16]. In brief, all surviving adults born in 1921, who turned 85 in 2006 when the study commenced and were permanently registered with a participating general practice in Newcastle upon Tyne or North Tyneside (North-East England) were invited to participate. People living in institutions and those with cognitive impairment were included. Participants were visited by a research nurse at their usual place of residence (i.e. their own home, care home, nursing home or residential home) at baseline (phase 1: 2006–7, n = 854), 18 months (phase 2: 2007–9, n = 631) and 36 months (phase 3: 2009–10, n = 484). Attrition between phases 1 and 3 was mainly due to deaths (62.7%, 232/370) with the remainder due to drop out. The study population at baseline was sociodemographically representative of the local (Newcastle upon Tyne and North Tyneside) and wider (England and Wales) population [15]. At each phase, a multi-dimensional health assessment was undertaken including questionnaires, physical measurements, function tests and blood sample measurements. Data reported here are from the phase 3 assessment (participants aged 88–89 years) unless otherwise stated. Control blood samples from anonymous volunteers were also collected for experimental analysis. Ethical approval was obtained from the Newcastle and North Tyneside 1 Research Ethics Committee (reference number 06/Q0905/2); written informed consent was obtained from either participants or from a consultee, usually a relative or carer, when participants lacked capacity to consent.

### ROS production and mitochondrial function measurements by flow cytometry

PBMCs from 6 ml lithium-heparin blood samples were isolated by density gradient centrifugation. Around 1×10^5^ cells were resuspended in 1 ml of RPMI medium (Sigma, Poole, UK) with either 10 µM dihydroethidium (DHE) (Life Technologies, Invitrogen, Paisly, UK) to measure superoxide levels, 100 nM MitoTracker Green FM (Life Technologies, Invitrogen, Paisly, UK) to measure mitochondrial mass or 7.7 µM 5,5′,6,6′-tetrachloro-1,1′,3,3′-tetraethylbenzimidaz olylcarbocyanine iodide (JC-1) (Life Technologies, Invitrogen, Paisly, UK) to measure mitochondrial membrane potential and incubated at 37°C for 30 minutes. RPMI medium alone was used as an unstained control. After incubation, cells were washed once and finally resuspended in 2 ml of RPMI medium. Samples were assessed immediately by flow cytometry on a Partec PAS system (Partec, Munster, Germany). Around 1×10^4^ events (cells) were analysed per sample. PBMCs, lymphocytes and monocytes were first identified based on their size and granularity [17] (Figure S1A.i, B.i and C.i in File S1). The mean fluorescence of unstained cells was recorded and subtracted from the fluorescence values measured in the stained cells, identified using the same gating as for the unstained cells. To confirm that the unstained cell population were red blood cells, and therefore correctly excluded, red blood cells were lysed before staining in a separate experiment (Figure S1A.ii, B.ii and C.ii in File S1). This was also confirmed by staining unlysed and lysed samples with 2 µl PerCP Mouse Anti-Human CD45 to identify PBMCs (lymphocytes sand monocytes) (Pharmingen, BD Biosciences, San Jose, CA, USA) in 1 ml RPMI medium (Figure S1D.i. and ii in File S1 respectively). Correct distinction between lymphocytes and monocytes by size/granularity was confirmed with a parallel experiment performed in a subset of control samples where 1×10^5^ cells were incubated with 2 µl PerCP Mouse Anti-Human CD45 in 1 ml RPMI medium to identify both lymphocytes and monocytes and 6 µl FITC Mouse Anti-Human CD14 (Pharmingen, BD Biosciences, San Jose, CA, USA) in 1 ml RPMI medium to identify monocytes only (Figure S1E in File S1).

### Other biomarkers measured in the Newcastle 85+ Study

Other biomarkers included in this analysis were those considered as potential markers of oxidative stress-induced cellular senescence (see Figure 1) including: plasma 8-isoprostanes as a marker of oxidative stress; PBMC telomere length as a marker of telomere shortening; ionized radiation-induced DNA damage and repair capacity of PBMCs as markers of DNA damage and repair; immunosenescence of PBMC subpopulations (as percentage of CD27-/RO- CD4 and CD8 T lymphocyte cells) as markers of cellular senescence; and interleukin-6 (IL-6), tumour necrosis factor-alpha (TNF-α) and high sensitivity C-reactive protein (hsCRP) as markers of inflammation. Additional physical, physiological, haematological and biochemical measurements that were previously confirmed as informative BoA in the Newcastle 85+ study [18] also included were: hand grip strength, timed up and go test (TUG), lung forced expiratory volume in 1 second (FEV_1_) (at phase 1), systolic blood pressure, haematocrit, haemoglobin, red blood cell count, free triiodothyronine (Free T3), vitamin D (at phase 1) and N-terminal pro b-type natriuretic peptide (NT-pro BNP). Details of the methology for all of these biomarkers have been reported [18] and are given as supplementary methods in File S1.

### Age-related outcomes

The procedures for the evaluation of cognitive impairment, disability score and count of chronic diseases have been reported [15]. Survival was calculated from date of biomarker measurement to death or censored at 30/04/2012.

### Statistical Analysis

For experimental data, the Student's t-Test was used to test differences between two samples, the coefficient of variation (CV, %) was used to assess assay precision and Pearson's correlation coefficient was used to test the strength of association between two continuous variables. Superoxide levels, mitochondrial mass and mitochondrial membrane potential each had a non-normal distribution in the study population, assessed using the Kolomogorov-Smirnov test for normality (p<0.05). We therefore used Spearman's correlation to test for the relationship between two continuous variables, Mann-Whitney U to test for the association between two groups and Kruskal-Wallis to test for the association between three or more groups. To test associations with survival, participants were divided into quartiles of superoxide levels, mitochondrial mass and mitochondrial membrane potential with the middle two quartiles grouped together to form the reference category to represent medium levels. Cox regression was used to test for the association between grouped variables and survival. p-values <0.05 were considered statistically significant. Since many of the markers are expected to have strong correlations with each other, reflecting common biological mechanisms, we did not apply a formal statistical correction for multiple comparisons since this would have been over-conservative. Sensitivity analysis was carried out by removing extreme outliers which were values more than 3 times the interquartile range (IQR) below the 25^th^ or above the 75^th^ percentiles, which were identified by default in SPSS boxplots. Potential confounders investigated were gender, marital status, housing, having had any higher education and physical activity. Data analysis was performed using SPSS version 19.0.

## Results

### Reliability of ROS production and mitochondrial function measurements in PBMCs

To ensure that PBMC superoxide levels, mitochondrial mass and mitochondrial membrane potential remained stable between sample handling and flow cytometry analysis, various factors that may affect their stability were investigated in control samples (Table S1 in File S1). There was no significant effect after 2 hours storage of samples at 37°C prior to staining for any of the parameters, but after 24 hours of storage at 37°C a significant increase of DHE signals could be observed (p<0.01), independent of mitochondrial mass and mitochondrial membrane potential. All parameters were affected by frozen storage of cells in 90% foetal bovine serum and 10% dimethyl sulfoxide. Following storage at −70°C, DHE signals increased (p<0.01) and the JC1 ratio indicating mitochondrial membrane potential decreased (p = 0.02), however there was no change in the mitotracker signal. Following storage at −196°C, DHE and mitotracker signal intensities increased while the JC1 ratio decreased (p<0.01). These changes might reflect damage to the cells during freezing/thawing. There was no significant effect of vortex-mediated mechanical stress for any of the parameters. Removing red blood cells by lysing them significantly stressed the remaining PBMCs as evidenced by increased superoxide levels and decreased mitochondrial membrane potential (p = 0.01). There was no significant effect on repeatedly freeze-thawing DHE, MitoTracker Green FM or JC-1 stain solutions before cell staining on all parameters for up to 10 cycles. There was no effect of light exposure on stained cells (i.e. stained samples were either light protected with foil or light exposed) but there was a significant effect of staining temperature on all parameters.

After considering the above experimental variables, the reliability of our flow cytometry method to evaluate ROS production and mitochondrial function in PBMCs and subpopulations was assessed by calculating the average intra- and inter-assay CV of three individual control samples of the same age. The intra- and inter-assay precision of all parameters in all cell subpopulations was acceptable [19]; superoxide levels measured by DHE showed an intra- and inter-assay CVs of 5–7%, mitochondrial mass measured by MitoTracker Green FM showed intra-assay CV of 3–10% and inter-assay CV of 11–13% and mitochondrial membrane potential measured by JC-1 showed intra-assay CV of 6–12% and inter-assay CV of 11–22%.

To evaluate the intra-individual stability of the parameters and the day-day reproducibility of our whole experimental procedure, 6 individual control blood samples were taken on two occasions with an interval of one week and samples were processed independently (Figure S2 in File S1). Measurements of superoxide levels and mitochondrial mass in total PBMCs and lymphocytes were strongly correlated at both time points (r≥0.66) and showed acceptable CVs (4%–8%); however measurements in monocytes showed little correlation (r≤0.19) despite acceptable CVs (12% and 13%). Measurements of mitochondrial membrane potential strongly correlated in PBMCs and lymphocytes (r≥0.64) but showed large CVs between time points (39% and 24%) and measurement in monocytes showed little correlation between time points (r = 0.25) and also a large CV (57%). Since all the parameters were unstable in the monocyte subpopulation, further measurement of the parameters in monocytes were excluded.

### Analysis of ROS production and mitochondrial function within the Newcastle 85+ study

The experimental variables found to affect ROS production and mitochondrial function in the control samples were considered in the measurements conducted within the Newcastle 85+ cohort to avoid measurement error (i.e. all samples included were stored for less then 24 hours, were not frozen before analysis, were not subject to red blood cell lysis and were stained at strictly 37°C for 30 minutes). Descriptive statistics of superoxide levels, mitochondrial mass and mitochondrial membrane potential as well as other biomarkers measured within this study are shown in Table 1. Superoxide levels were significantly associated with mitochondrial mass (positive in PBMCs: p = 0.04; positive in lymphocytes: p = 0.01) and mitochondrial membrane potential (negative in PBMCs, p = 0.01; positive in lymphocytes, p = 0.04) (Table S2 and Figure S3 in File S1). Sensitivity analysis removed the significance of the association between superoxide levels and mitochondrial mass in the PBMC population (p = 0.13), however the significance still remained in the lymphocyte subpopulation (p = 0.01) (Table S3 in File S1). All other significant correlations and direction of associations remained after sensitivity analysis. Superoxide levels, mitochondrial mass and mitochondrial membrane potential were not associated with gender, marital status, housing, having had any higher education or physical activity (data not shown).

**Table 1:** Descriptive statistics of biomarker measurements.

	Descriptives				
Biomarker	Mean	SE	Median	IQR	n
**ROS production and mitochondrial dysfunction**					
Superoxide levels (AU)					
*PBMCs*	37.99	1.15	33.94	20.17	248
*Lymphocytes*	29.39	1.11	25.39	20.81	248
Mitochondrial mass (AU)					
*PBMCs*	43.35	0.89	41.69	19.99	341
*Lymphocytes*	51.43	1.75	44.6	32.06	341
Mitochondrial membrane potential (AU)					
*PBMCs*	3.16	0.07	2.86	0.98	347
*Lymphocytes*	4.24	0.10	3.85	2.00	347
**Oxidative stress**					
8-Iso Prostaglandin F_2α_ (ng/ml)	2.69	0.16	1.49	2.08	379
**Telomere shortening**					
Telomere length (bp)	2825.03	37.55	2715.77	1057.17	430
**DNA damage and repair**					
DNA damage (%)	46.22	0.91	47.26	29.65	431
DNA repair (%)	48.47	1.35	46.24	47.27	431
**Immunosenescence**					
CD27-/RO- CD4 T lymphocytes (%)	4.10	0.35	1.46	3.32	429
CD27-/RO- CD8 T lymphocytes (%)	24.66	0.85	21.81	25.69	403
**Inflammation**					
Il-6 (pg/ml)	539.55	133.85	10.25	31.43	430
TNF-α (pg/ml)	37.64	9.86	2.55	4.42	430
hsCRP (mg/l)	5.63	0.63	2.40	3.70	434
**Informative BoA**					
Hand grip strength (kg)	17.06	0.35	16.10	9.03	453
TUG (seconds)	21.52	0.94	17.09	12.19	402
FEV_1_ (l)^P1^	144.55	1.94	137.00	71.75	776
Systolic blood pressure (mmHg)	143.04	0.96	144.00	28.00	462
Haematocrit (%)	0.39	0.00	0.39	0.05	427
Haemoglobin (g/dl)	12.90	0.07	13.00	1.70	427
Red blood cells (x 10^12^/l)	4.25	0.02	4.25	0.62	427
Free T3 (pmol/l)	4.39	0.03	4.40	0.80	426
Vitamin D (nmol/l)^P1^	45.36	0.94	39.00	37.00	778
NT-pro BNP (pg/ml)	848.21	89.05	514.00	791.75	124

(SE: Standard error, IQR: Interquartile range, n: Number of participants, ^P1^Baseline (phase 1)).

We next tested the association between ROS production and mitochondrial function with other biomarkers considered as potential markers of oxidative stress-induced cellular senescence (see Figure 1). There were consistent associations (i.e. for more than one cell population) between mitochondrial mass and telomere length (positive, p≤0.04), between superoxide levels and DNA repair activity (negative, p≤0.02) and between superoxide levels and levels of the pro-inflammatory cytokines Il-6 and TNF-α (negative, p<0.01) (Table 2 and Figure S4 in File S1). Sensitivity analysis by removing extreme outliers did not affect the significance or the direction of association (Table S4 and Figure S5 in File S1).

**Table 2:** Superoxide levels, mitochondrial mass and mitochondrial membrane potential in relation to other potential markers of oxidative stress induced cellular senescence.

		Superoxide levels		Mitochondrial mass		Mitochondrial membrane potential	
		*PBMCs*	*Lymphocytes*	*PBMCs*	*Lymphocytes*	*PBMCs*	*Lymphocytes*
**8-iso Prostaglandin F_2_**_α_	r	−0.05	−0.03	0.06	0.03	0.03	0.02
	p	0.45	0.65	0.34	0.64	0.62	0.77
	n	216	216	302	302	304	304
**Telomere length**	r	0.11	0.08	**0.11**	**0.13**	**0.13**	0.04
	p	0.08	0.23	**0.04**	**0.02**	**0.01**	0.41
	n	248	248	**340**	**340**	**345**	345
**DNA damage**	r	−0.06	−0.09	0.05	−0.03	0.03	0.03
	p	0.33	0.16	0.38	0.53	0.63	0.64
	n	248	248	341	341	347	347
**DNA repair**	r	**−0.16**	**−0.15**	−0.06	−0.07	0.06	−0.03
	p	**0.01**	**0.02**	0.26	0.21	0.24	0.59
	n	**248**	**248**	341	341	347	347
**CD27-/RO- CD4 T lymphocytes**	r	−0.11	−0.07	0.08	0.08	0.04	−0.01
	p	0.09	0.31	0.15	0.13	0.42	0.91
	n	246	246	339	339	344	344
**CD27-/RO- CD8 T lymphocytes**	r	−0.09	−0.04	0.09	0.08	0.03	−0.02
	p	0.17	0.57	0.12	0.14	0.62	0.75
	n	237	237	317	317	323	323
**Il-6**	r	**−0.31**	**−0.32**	−0.02	−0.05	−0.07	**−0.15**
	p	**0.00**	**0.00**	0.72	0.31	0.23	**0.00**
	n	**247**	**247**	340	340	345	**345**
**TNF**-α	r	**−0.23**	**−0.23**	−0.03	−0.05	−0.03	**−0.11**
	p	**0.00**	**0.00**	0.60	0.33	0.58	**0.04**
	n	**247**	**247**	340	340	345	**345**
**hsCRP**	r	0.08	0.11	0.03	0.00	−0.10	−0.01
	p	0.19	0.08	0.56	0.94	0.05	0.81
	n	248	248	341	341	347	347

(r: Spearman's correlation coefficient, p: probability, n: number of participants).

Previously we had confirmed 10 out of a list of 72 candidate markers as informative BoA in the Newcastle 85+ study because they were associated with two or more of the following age-related outcomes: cognitive impairment, disability score, disease count and survival [18]. We therefore tested the association between ROS production and mitochondrial function with the 10 informative BoA (Table 3 and Figure S6 in File S1). No consistent associations (i.e. for more than one cell population) were found however significant associations were found in lymphocytes between Free T3 with mitochondrial membrane potential (p = 0.03) and baseline vitamin D levels with superoxide levels (p = 0.04) and mitochondrial membrane potential (p<0.01). Sensitivity analysis by removing extreme outliers reduced the significance of the association between superoxide levels and vitamin D in the lymphocyte subpopulation (p = 0.08) however the association between Free T3 and vitamin D with mitochondrial membrane potential in the lymphocyte subpopulation remained (p≤0.03) (Table S5 and Figure S7 in File S1).

**Table 3:** Superoxide levels, mitochondrial mass and mitochondrial membrane potential in relation to informative BoA.

		Superoxide levels		Mitochondrial mass		Mitochondrial membrane potential	
		*PBMCs*	*Lymphocytes*	*PBMCs*	*Lymphocytes*	*PBMCs*	*Lymphocytes*
**Hand grip strength**	r	0.01	0.00	−0.02	−0.05	−0.06	−0.01
	p	0.85	0.95	0.71	0.35	0.29	0.87
	n	242	242	336	336	342	342
**TUG**	r	−0.01	−0.01	0.03	0.05	0.06	0.07
	p	0.94	0.91	0.64	0.37	0.30	0.23
	n	218	218	304	304	312	312
**FEV1^P1^**	r	0.07	0.06	0.02	−0.04	0.01	−0.01
	p	0.29	0.32	0.77	0.49	0.85	0.83
	n	244	244	335	335	341	341
**Systolic blood pressure**	r	0.02	−0.02	0.07	0.09	0.01	0.01
	p	0.77	0.79	0.22	0.09	0.92	0.87
	n	245	245	338	338	343	343
**Haematocrit**	r	0.06	0.07	0.05	−0.03	−0.04	−0.05
	p	0.37	0.26	0.35	0.53	0.49	0.36
	n	246	246	338	338	343	343
**Haemoglobin**	r	0.05	0.07	0.08	−0.01	0.01	−0.06
	p	0.44	0.25	0.16	0.81	0.82	0.29
	n	246	246	338	338	343	343
**Red blood cells**	r	0.08	0.12	0.10	0.05	−0.03	−0.08
	p	0.20	0.07	0.08	0.36	0.6	0.13
	n	246	246	338	338	343	343
**Free T3**	r	−0.09	−0.11	0.03	−0.04	−0.07	**−0.12**
	p	0.16	0.08	0.61	0.51	0.19	**0.03**
	n	246	246	338	338	343	**343**
**Vitamin D^P1^**	r	−0.12	**−0.13**	−0.01	−0.05	−0.10	**−0.18**
	p	0.06	**0.04**	0.89	0.35	0.06	**00.0**
	n	240	**240**	331	331	338	**338**
**NT-pro BNP**	r	−0.10	−0.19	−0.09	−0.17	−0.10	0.11
	p	0.50	0.19	0.39	0.12	0.33	0.30
	n	46	46	85	85	95	95

(r: Spearman's correlation coefficient, p: probability, n: number of participants, ^P1^Phase 1 data).

**Figure 2:**
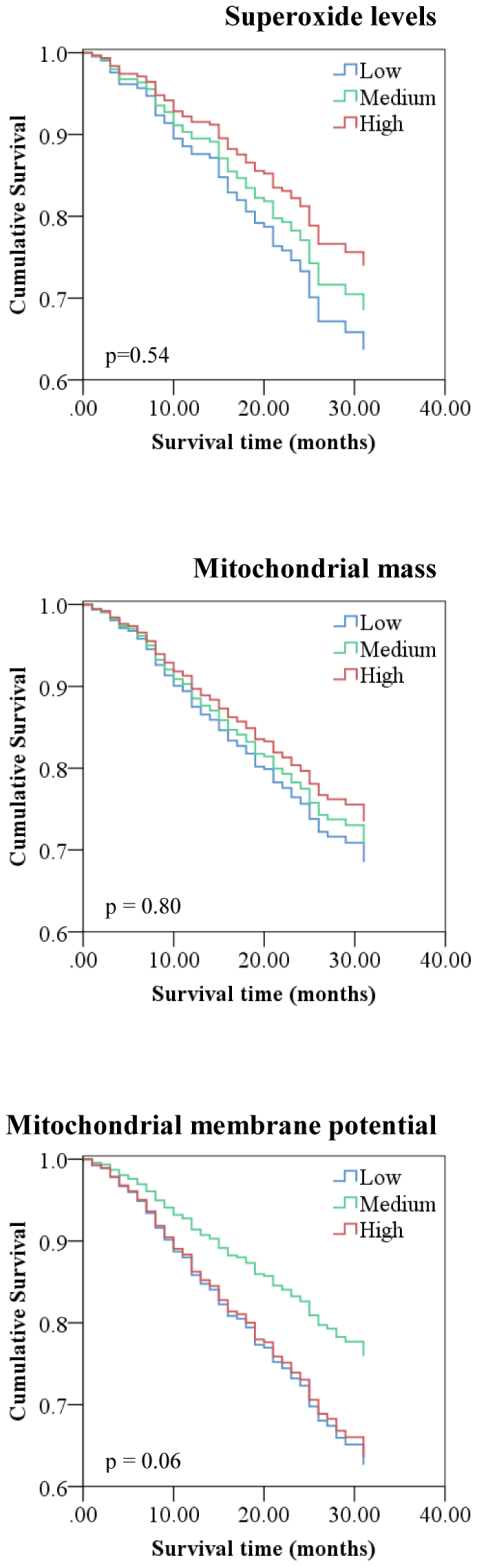
Association between PBMC superoxide levels, mitochondrial mass, mitochondrial membrane potential and survival. (Cox regression analysis)

The association between ROS production and mitochondrial function and age-related outcomes including cognitive impairment, disability and count of chronic diseases (Table 4) and survival (Figure 2) in this sample of the very old was investigated. No significant associations were found between superoxide levels or the mitochondrial parameters with cognitive impairment, disability, disease count or survival. This was not changed following removal of outliers (Table S6 in File S1).

**Table 4:** Association between superoxide levels, mitochondrial mass, mitochondrial membrane potential and age-related outcomes.

		Superoxide levels		Mitochondrial mass		Mitochondrial membrane potential	
		*PBMCs*	*Lymphocytes*	*PBMCs*	*Lymphocytes*	*PBMCs*	*Lymphocytes*
**Disability score**	r	0.00	0.01	−0.03	0.01	0.02	0.07
	p	0.95	0.90	0.60	0.83	0.75	0.21
	n	247	247	339	339	345	345
**SMMSE score**	r	0.07	0.08	0.08	0.04	0.05	−0.08
	p	0.25	0.19	0.13	0.44	0.35	0.13
	n	247	247	339	339	345	345
**Disease count**	r	0.08	0.08	0.04	0.07	−0.03	−0.01
	p	0.20	0.25	0.46	0.22	0.55	0.90
	n	239	239	328	328	334	334

(r: Spearman's correlation coefficient, p: probability, n: number of participants).

## Discussion

We provide evidence for the methodological reliability of measuring superoxide levels, mitochondrial mass and mitochondrial membrane potential by flow cytometry in PBMCs and lymphocyte subpopulations. We did not however find evidence for their validity as predictors of age-related health outcomes or survival in the very old.

Assessing the reliability of potential BoA is critically important in population studies to ensure that variability reflects genuine inter-individual differences and not methodological error. This study aimed to evaluate measurement error by investigating variables that may affect the experimental stability of ROS production and mitochondrial function as potential BoA. Variables found to affect the stability of the markers in PBMCs during preparation before flow cytometry analysis were: sample storage at 37°C for 24 hours; freezing at −70°C and −196°C; removing red blood cells by lysis; and the staining temperature of cells. Short term sample storage (37°C for 2 hours), vortex mediated mechanical stress, stain freeze-thaw cycles (up to 10 cycles) and not protecting samples from light exposure during staining did not influence the markers. The study also showed that all markers were stable within individuals during a seven day interval in the PBMC and lymphocyte populations but not in the monocyte population. It is therefore unlikely that intra-individual factors will contribute to measurement error in the short term within the two former cell populations however this could be the case within the monocyte population. In support of this, monocyte populations have been shown to have a weaker diurnal rhythm than lymphocytes and other PBMC subpopulations which demonstrates that they may be more sensitive to intra-individual factors [20], which could affect ROS production and mitochondrial function. One potential intra-individual factor is sleep quality since different PBMC subpopulation levels have different sensitivities to sleep deprivation [20]. Other potential causes of intra-individual variation that could be explored in future analysis include diet, smoking, alcohol consumption, medications, illnesses and other key events. The seven day interval experiment also demonstrated good day-day repeatability of the flow cytometry analysis for superoxide and mitochondrial mass but not for mitochondrial membrane potential. A potential problem with the JC-1 dye for measuring mitochondrial membrane potential is that it is poorly soluble in aqueous solutions [21] and thus variation in the preparation of the stain on different occasions could be the reason for its day-day variability.

We have previously found in human fibroblasts, and in a number of other tissues, that high mitochondrial ROS production, high mitochondrial mass and low mitochondrial membrane potential are associated with cell senescence [22]–[26]. While the association of high mitochondrial mass with telomere dysfunction and senescence has been contested [27], increases in ROS and decreases in mitochondrial functionality, typically associated with low mitochondrial membrane potential, have commonly been found in ageing and senescence [28]–[31]. We therefore expected to find robust associations between ROS production and mitochondrial dysfunction with a range of other senescence-associated parameters in the peripheral blood samples from the Newcastle 85+ study. This was not the case; typically most correlations were either absent or opposed to the expected direction. Although superoxide levels and mitochondrial membrane potential were negatively associated in PBMCs, as seen in senescent fibroblasts [23], this association was reversed in lymphocytes. This could potentially be due to a non-linear association between ROS production and mitochondrial membrane potential. It might also suggest that ROS levels observed in lymphocytes from the Newcastle 85+ study participants are not primarily driven by cell senescence.

This study also attempted to validate superoxide levels and mitochondrial dysfunction as BoA by determining their agreement with 10 informative BoA identified in a previous investigation of the Newcastle 85+ study cohort [18]. Increased mitochondrial membrane potential in Phase 3 lymphocytes significantly correlated with decreased serum Vitamin D levels in Phase 1. Although it has been shown there is a moderate intra-individual variation in vitamin D over approximately five years, a high correlation was observed, supporting the use of a one-time measurement of vitamin D within 5 years [32]. We would therefore expect vitamin D levels at phase 3 to show the same association to mitochondrial membrane potential as phase 1 levels. However vitamin D supplementation and season of blood draw affects the reliability of repeat measures. The association between mitochondrial membrane potential and vitamin D levels could therefore be stronger if we had measurements at the same time point. This could be worth further investigation. Mitochondrial membrane potential also significantly correlated with free T3. Since vitamin D levels and thyroid hormones vary with the season in the older population [33], this suggests that mitochondrial function could also be affected by seasonality. There were no other associations found between superoxide levels or mitochondrial parameters and the other eight remaining informative BoA.

Further attempts to validate ROS production and mitochondrial parameters as BoA were made by investigating their association with age-related outcomes in the very old, as in the previously reported study of this cohort by Martin-Ruiz et al, 2011. No associations were found between ROS production or the mitochondrial parameters with cognitive impairment, disability score, disease count or survival. However, since the very old have a high prevalence of chronic conditions, including hypertension, osteoarthritis and sensory impairment [15], this study could be confounded by the presence of other age-related outcomes. A more general approach could be to investigate the association between potential BoA and frailty. A previous analysis in the Newcastle 85+ study identified the importance of inflammatory markers in frailty in the oldest old [34]. However limited evidence was found for the role of immunosenescence in frailty and no evidence for the role of telomere length, markers of oxidative damage or DNA damage and repair. Furthermore, a recent analysis in the same cohort found no association between frailty and mitochondrial DNA haplogroups [35]. These are maternally inherited variations in mitochondrial DNA (mtDNA) sequences for which the population can be divided into several groups based on specific nucleotide polymorphisms (SNPs) variants. It is thought that some variants of mitochondrial DNA haplogroups have functional differences including lower rates of ROS production and better capacity to cope with oxidative stress [36], [37] and several studies have shown associations with various age-related outcomes [38]–[41]. However there are inconsistencies regarding which variant is more susceptible to age-related outcomes. A limitation, along with this study in relation to cognitive impairment, disability score and disease count, is that analysis was cross-sectional and longitudinal analysis would be more informative. Further analysis of this cohort could therefore be to longitudinally investigate the role of ROS production and mitochondrial function in the development of frailty within the very old.

## Conclusion

This study identified variables that affect the stability of PBMC ROS production and mitochondrial function during experimental analysis and provided evidence of their experimental reliability and intra-individual stability in PBMC and lymphocyte subpopulations. The reason for the individual instability in monocytes should be further explored. We also provided evidence for the agreement of PBMC ROS production and mitochondrial function with some markers implicated in oxidative stress-induced cellular senescence, however some associations were in the opposite direction to those expected. We do not however provide evidence for their validity as markers associated with age-related outcomes or survival, and therefore as candidate BoA in the very old population.

## Supporting Information

File S1 **File S1 includes the following: Figure S1.** Quantification of superoxide levels, mitochondrial mass and mitochondrial membrane potential in PBMCs and cell subpopulations by flow cytometry. **Figure S2**. Short term intra-individual stability and day-day precision of superoxide levels, mitochondrial mass and mitochondrial membrane potential. **Figure S3**. Scatter plots of agreements between superoxide levels, mitochondrial mass and mitochondrial membrane potential. **Figure S4**. Scatter plots of superoxide levels, mitochondrial mass and mitochondrial membrane potential in relation to other potential markers of oxidative stress-induced cellular senescence. **Figure S5**. Scatter plots of superoxide levels, mitochondrial mass and mitochondrial membrane potential in relation to other potential markers of oxidative stress-induced cellular senescence after removal of extreme outliers. **Figure S6**. Scatter plots of superoxide levels, mitochondrial mass and mitochondrial membrane potential in relation to informative BoA. **Figure S7**. Scatter plots of superoxide levels, mitochondrial mass and mitochondrial membrane potential in relation to informative BoA after removal of extreme outliers. **Table S1**. Stability of superoxide levels, mitochondrial mass and mitochondrial membrane potential in PBMCs during various experimental handling. **Table S2**. Agreements between superoxide levels, mitochondrial mass and mitochondrial membrane potential. **Table S3**. Agreements between superoxide levels, mitochondrial mass and mitochondrial membrane potential after removal of extreme outliers. **Table S4**. Superoxide levels, mitochondrial mass and mitochondrial membrane potential in relation to other potential markers of oxidative stress-induced cellular senescence after removal of extreme outliers. **Table S5**. Superoxide levels, mitochondrial mass and mitochondrial membrane potential in relation to informative BoA after removal of extreme outliers. **Table S6**. Association between superoxide levels, mitochondrial mass and mitochondrial membrane potential and age-related outcomes after removal of extreme outliers. **Supplementary Methods**.
(DOCX)

## References

[1] Office-for-National-Statistics (2011) UK Interim Life Tables, 1980–82 to 2008–10.

[2] Office-for-National-Statistics (2010) United Kingdom Health Statistics 2010.

[3] Kirkwood TBL (2008) A systematic look at an old problem. Nature 451: 644–647. 18256658 10.1038/451644a

[4] Kirkwood TBL (1998) Alex Comfort and the measure of aging. Exp Gerontol 33: 135–140. 9467723 10.1016/s0531-5565(97)00114-9

[5] Sprott RL (2010) Biomarkers of aging and disease: Introduction and definitions. Exp Gerontol 45: 2–4. 19651201 10.1016/j.exger.2009.07.008

[6] Droge W (2002) Free radicals in the physiological control of cell function. Physiol Rev 82: 47–95. 11773609 10.1152/physrev.00018.2001

[7] Boveris A, Chance B (1973) The mitochondrial generation of hydrogen peroxide. General properties and effect of hyperbaric oxygen. Biochem J 134: 707–716. 4749271 10.1042/bj1340707 PMC1177867

[8] Richter C, Park JW, Ames BN (1988) Normal oxidative damage to mitochondrial and nuclear DNA is extensive. Proc Natl Acad Sci U S A 85: 6465–6457. 3413108 10.1073/pnas.85.17.6465 PMC281993

[9] Batandier C, Leverve X, Fontaine E (2004) Opening of the mitochondrial permeability transition pore induces reactive oxygen species production at the level of the respiratory chain complex I. J Biol Chem. 279: 17197–17204. 10.1074/jbc.M310329200 14963044

[10] Zorov DB, Filburn CR, Klotz LO, Zweier JL, Sollott SJ (2000) Reactive oxygen species (ROS)-induced ROS release: A new phenomenon accompanying induction of the mitochondrial permeability transition in cardiac myocytes. J Exp Med 192: 1001–1014. 11015441 10.1084/jem.192.7.1001 PMC2193314

[11] Nesselroade JR, Ram N (2004) Studying Intraindividual Variability: What We Have Learned That Will Help Us Understand Lives in Context. Res Hum Dev 1: 9–29.

[12] Mayeux R (2004) Biomarkers: potential uses and limitations. NeuroRx 1: 182–188. 15717018 10.1602/neurorx.1.2.182 PMC534923

[13] Martin-Ruiz C, von Zglinicki T, editors (2013) A life-course approach to biomarkers of ageing : Oxford University Press(in press).

[14] Collerton J, Barrass K, Bond J, Eccles M, Jagger C, et al (2007) The Newcastle 85+ study: biological, clinical and psychosocial factors associated with healthy ageing: study protocol. BMC Geriatr 7: 1–7. 17594470 10.1186/1471-2318-7-14 PMC1924857

[15] Collerton J, Davies K, Jagger C, Kingston A, Bond J, et al (2009) Health and disease in 85 year olds: baseline findings from the Newcastle 85+ cohort study. BMJ 339: 1–11. 10.1136/bmj.b4904 PMC2797051 20028777

[16] Davies K, Collerton JC, Jagger C, Bond J, Barker SA, et al (2010) Engaging the oldest old in research: lessons from the Newcastle 85+ study. BMC Geriatr 10: 1–9. 20849598 10.1186/1471-2318-10-64 PMC2945353

[17] Fleisher TA, Marti GE (2001) Detection of unseparated human lymphocytes by flow cytometry. Curr Protoc Immunol Chapter 7 : Unit 7.9. 10.1002/0471142735.im0709s08 18432849

[18] Martin-Ruiz C, Jagger C, Kingston A, Collerton J, Catt M, et al (2011) Assessment of a large panel of candidate biomarkers of ageing in the Newcastle 85+ study. Mech Ageing Dev 132: 496–502. 21864562 10.1016/j.mad.2011.08.001

[19] Litwin V, Marder P, editors (2001) Flow cytometry in drug discovery and development. Hoboken, New Jersey: John Wiley & Sons. 235 p.

[20] Ackermann K, Revell VL, Lao O, Rombouts EJ, Skene DJ, et al (2012) Diurnal rhythms in blood cell populations and the effect of acute sleep deprivation in healthy young men. Sleep 35: 933–940. 22754039 10.5665/sleep.1954 PMC3369228

[21] Schwartz LM, Ashwell JD (2001) Dissipation of mitochondrial transmembrane potential during apoptosis. Methods Cell Biol 66: 82.

[22] Passos JF, Nelson G, Wang CF, Richter T, Simillion C, et al (2010) Feedback between p21 and reactive oxygen production is necessary for cell senescence. Mol Syst Biol 6: 1–9. 10.1038/msb.2010.5 PMC2835567 20160708

[23] Passos JF, Saretzki G, Ahmed S, Nelson G, Richter T, et al (2007) Mitochondrial dysfunction accounts for the stochastic heterogeneity in telomere-dependent senescence. PLoS Biol 5: e110. 17472436 10.1371/journal.pbio.0050110 PMC1858712

[24] Passos JF, von Zglinicki T, Kirkwood TB (2007) Mitochondria and ageing: winning and losing in the numbers game. Bioessays 29: 908–917. 17688237 10.1002/bies.20634

[25] Wang C, Jurk D, Maddick M, Nelson G, Martin-Ruiz C, et al (2009) DNA damage response and cellular senescence in tissues of aging mice. Aging Cell 8: 311–323. 19627270 10.1111/j.1474-9726.2009.00481.x

[26] Wang C, Maddick M, Miwa S, Jurk D, Czapiewski R, et al (2010) Adult-onset, short-term dietary restriction reduces cell senescence in mice. Aging (Albany NY) 2: 555–566. 20844316 10.18632/aging.100196 PMC2984605

[27] Sahin E, Colla S, Liesa M, Moslehi J, Muller FL, et al (2011) Telomere dysfunction induces metabolic and mitochondrial compromise. Nature 470: 359–365. 21307849 10.1038/nature09787 PMC3741661

[28] Hagen TM, Yowe DL, Bartholomew JC, Wehr CM, Do KL, et al (1997) Mitochondrial decay in hepatocytes from old rats: membrane potential declines, heterogeneity and oxidants increase. Proc Natl Acad Sci U S A 94: 3064–3069. 9096346 10.1073/pnas.94.7.3064 PMC20322

[29] Hagen TM, Ingersoll RT, Lykkesfeldt J, Liu JK, Wehr CM, et al (1999) (R)-alpha-lipoic acid-supplemented old rats have improved mitochondrial function, decreased oxidative damage, and increased metabolic rate. FASEB J 13: 411–418. 9973329 10.1096/fasebj.13.2.411

[30] Kokoszka JE, Coskun P, Esposito LA, Wallace DC (2001) Increased mitochondrial oxidative stress in the Sod2 (+/−) mouse results in the age-related decline of mitochondrial function culminating in increased apoptosis. Proc Natl Acad Sci U S A 98: 2278–2283. 11226230 10.1073/pnas.051627098 PMC30129

[31] Moiseeva O, Bourdeau V, Roux A, Deschenes-Simard X, Ferbeyre G (2009) Mitochondrial Dysfunction Contributes to Oncogene-Induced Senescence. Mol Cell Biol 29: 4495–4507. 19528227 10.1128/MCB.01868-08 PMC2725737

[32] Meng JE, Hovey KM, Wactawski-Wende J, Andrews CA, Lamonte MJ, et al (2012) Intraindividual variation in plasma 25-hydroxyvitamin D measures 5 years apart among postmenopausal women. Cancer Epidemiol Biomarkers Prev 21: 916–924. 22523182 10.1158/1055-9965.EPI-12-0026 PMC3372646

[33] Houston DK, Neiberg RH, Tooze JA, Hausman DB, Johnson MA, et al (2013) Low 25-hydroxyvitamin D predicts the onset of mobility limitation and disability in community-dwelling older adults: the Health ABC Study. J Gerontol A Biol Sci Med Sci 68: 181–187. 22573914 10.1093/gerona/gls136 PMC3598356

[34] Collerton J, Martin-Ruiz C, Davies K, Hilkens CM, Isaacs J, et al (2012) Frailty and the role of inflammation, immunosenescence and cellular ageing in the very old: cross-sectional findings from the Newcastle 85+ Study. Mech Ageing Dev 133: 456–466. 22663935 10.1016/j.mad.2012.05.005

[35] Collerton J, Ashok D, Martin-Ruiz C, Pyle A, Hudson G, et al (2013) Frailty and mortality are not influenced by mitochondrial DNA haplotypes in the very old. Neurobiol Aging 34: 2699–2892. 23639206 10.1016/j.neurobiolaging.2013.04.001 PMC3906612

[36] Mueller EE, Brunner SM, Mayr JA, Stanger O, Sperl W, et al (2012) Functional Differences between Mitochondrial Haplogroup T and Haplogroup H in HEK293 Cybrid Cells. PLoS One 7: e52367. 23300652 10.1371/journal.pone.0052367 PMC3530588

[37] Chen A, Raule N, Chomyn A, Attardi G (2012) Decreased Reactive Oxygen Species Production in Cells with Mitochondrial Haplogroups Associated with Longevity. PLoS One 7: e46473. 23144696 10.1371/journal.pone.0046473 PMC3483264

[38] van der Walt JM, Dementieva YA, Martin ER, Scott WK, Nicodemus KK, et al (2004) Analysis of European mitochondrial haplogroups with Alzheimer disease risk. Neurosci Lett 365: 28–32. 15234467 10.1016/j.neulet.2004.04.051

[39] Ghezzi D, Marelli C, Achilli A, Goldwurm S, Pezzoli G, et al (2005) Mitochondrial DNA haplogroup K is associated with a lower risk of Parkinson's disease in Italians. Eur J Hum Genet 13: 748–752. 15827561 10.1038/sj.ejhg.5201425

[40] Wolf C, Gramer E, Muller-Myhsok B, Pasutto F, Wissinger B, et al. (2010) Mitochondrial haplogroup U is associated with a reduced risk to develop exfoliation glaucoma in the German population. BMC Genet 11.. 10.1186/1471-2156-11-8 PMC2834599 20109175

[41] Kofler B, Mueller EE, Eder W, Stanger O, Maier R, et al. (2009) Mitochondrial DNA haplogroup T is associated with coronary artery disease and diabetic retinopathy: a case control study. BMC Med Genet 10.. 10.1186/1471-2350-10-35 PMC2676278 19383124

[42] von Zglinicki T (2002) Oxidative stress shortens telomeres. Trends Biochem Sci 27: 339–344. 12114022 10.1016/s0968-0004(02)02110-2

[43] von Zglinicki T, Saretzki G, Docke W, Lotze C (1995) Mild hyperoxia shortens telomeres and inhibits proliferation of fibroblasts: a model for senescence? Exp Cell Res 220: 186–193. 7664835 10.1006/excr.1995.1305

[44] Nelson G, Wordsworth J, Wang C, Jurk D, Lawless C, et al (2012) A senescent cell bystander effect: senescence-induced senescence. Aging Cell 11: 345–349. 22321662 10.1111/j.1474-9726.2012.00795.x PMC3488292

[45] Tchkonia T, Morbeck DE, Von Zglinicki T, Van Deursen J, Lustgarten J, et al (2010) Fat tissue, aging, and cellular senescence. Aging Cell 9: 667–684. 20701600 10.1111/j.1474-9726.2010.00608.x PMC2941545

[46] Baker DJ, Wijshake T, Tchkonia T, LeBrasseur NK, Childs BG, et al (2011) Clearance of p16Ink4a-positive senescent cells delays ageing-associated disorders. Nature 479: 232–236. 22048312 10.1038/nature10600 PMC3468323

