# Supplementary material for: Reactive Oxygen Species Production and Mitochondrial Dysfunction in White Blood Cells Are Not Valid Biomarkers of Ageing in the Very Old
Source: PLoS One. 2014 Mar 10;9(3):e91005. doi: 10.1371/journal.pone.0091005 (PMC3948743; doi:10.1371/journal.pone.0091005)
Supplement: File S1 — includes the following: Figure S1. Quantification of superoxide levels, mitochondrial mass and mitochondrial membrane potential in PBMCs and cell subpopulations by flow cytometry. Figure S2. Short term intra-individual stability and day-day precision of superoxide levels, mitochondrial mass and mitochondrial membrane potential. Figure S3. Scatter plots of agreements between superoxide levels, mitochondrial mass and mitochondrial membrane potential. Figure S4. Scatter plots of superoxide levels, mitochondrial mass and mitochondrial membrane potential in relation to other potential markers of oxidative stress-induced cellular senescence. Figure S5. Scatter plots of superoxide levels, mitochondrial mass and mitochondrial membrane potential in relation to other potential markers of oxidative stress-induced cellular senescence after removal of extreme outliers. Figure S6. Scatter plots of superoxide levels, mitochondrial mass and mitochondrial membrane potential in relation to informative BoA. Figure S7. Scatter plots of superoxide levels, mitochondrial mass and mitochondrial membrane potential in relation to informative BoA after removal of extreme outliers. Table S1. Stability of superoxide levels, mitochondrial mass and mitochondrial membrane potential in PBMCs during various experimental handling. Table S2. Agreements between superoxide levels, mitochondrial mass and mitochondrial membrane potential. Table S3. Agreements between superoxide levels, mitochondrial mass and mitochondrial membrane potential after removal of extreme outliers. Table S4. Superoxide levels, mitochondrial mass and mitochondrial membrane potential in relation to other potential markers of oxidative stress-induced cellular senescence after removal of extreme outliers. Table S5. Superoxide levels, mitochondrial mass and mitochondrial membrane potential in relation to informative BoA after removal of extreme outliers. Table S6. Association between superoxide levels, mitochondrial mass and [file pone.0091005.s001.docx]

**Figure S1. Quantification of superoxide levels, mitochondrial mass and mitochondrial membrane potential in PBMCs and cell subpopulations by flow cytometry.** A. Superoxide levels: unstained cells and DHE stained cells in i. unlysed and ii. lysed samples, B. Mitochondrial mass: unstained cells and MitoTracker Green FM stained cells in i. unlysed and ii. lysed samples, C. Mitochondrial membrane potential: unstained and JC-1 stained cells in i. unlysed and ii. lysed samples, D. CD45 expression: unstained cells and PerCP Mouse Anti-Human CD45 stained cells in i. unlysed and ii. lysed samples E. Confirmation of PBMC subpopulations: unstained cells and PerCP Mouse Anti-Human CD45 and FITC Mouse Anti-Human CD14 stained cells. (●: Red blood cells (CD45-CD14-), ●: PBMCs (CD45+), ●: Lymphocytes (CD45+CD14-), ●: Monocytes (CD45+CD14+))


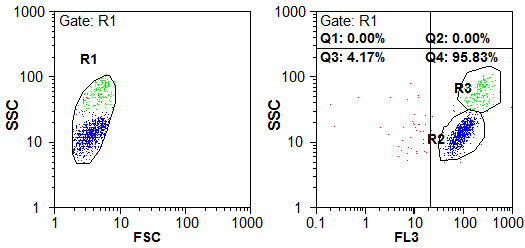

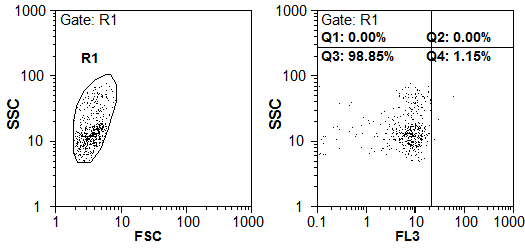

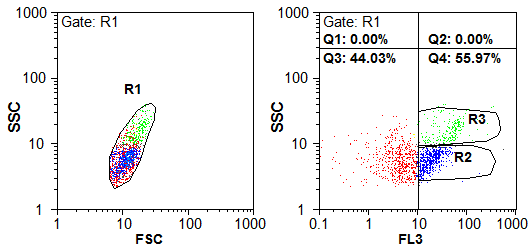

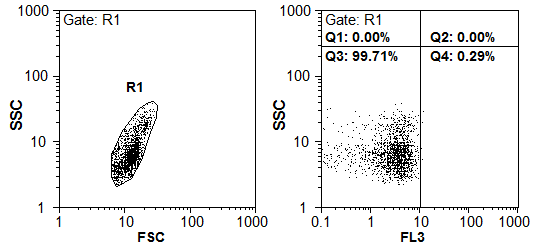


Unstained Unstained

DHE stained DHE stained


**i. Unlysed ii. Lysed**

Unstained Unstained

MitoTracker Green FM stained MitoTracker Green FM stained


**i. Unlysed ii. Lysed**


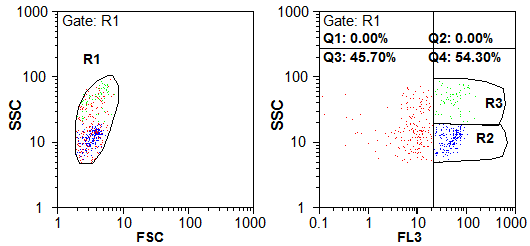

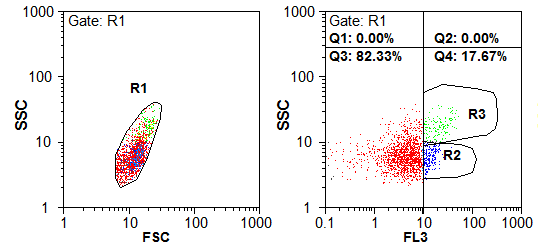

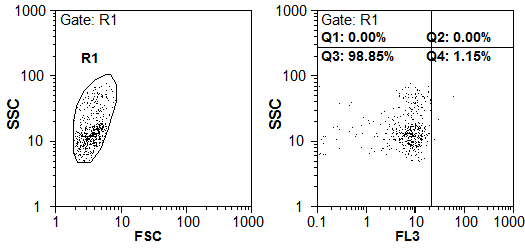

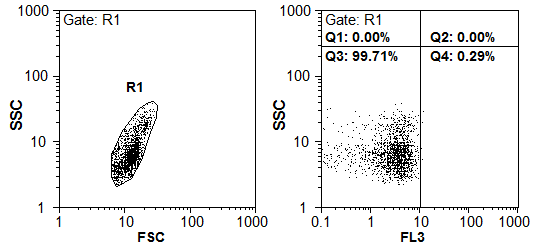

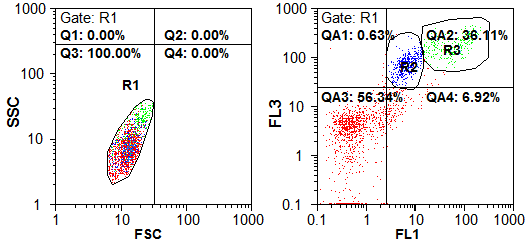

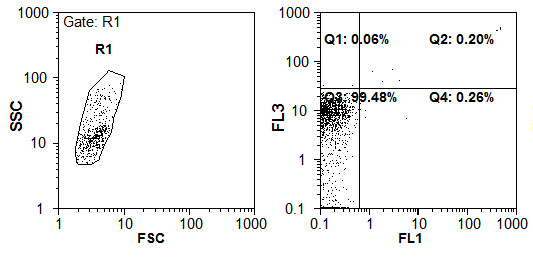

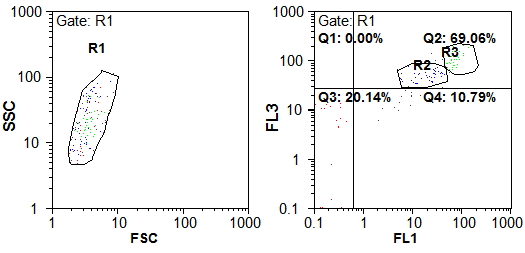

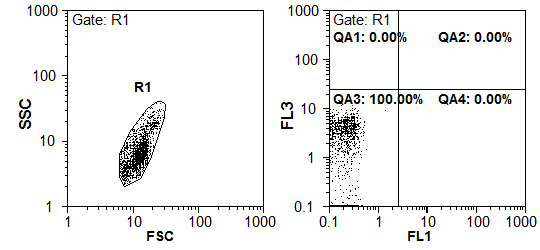


**i. Unlysed ii. Lysed**

Unstained Unstained

JC-1 stained JC-1 stained


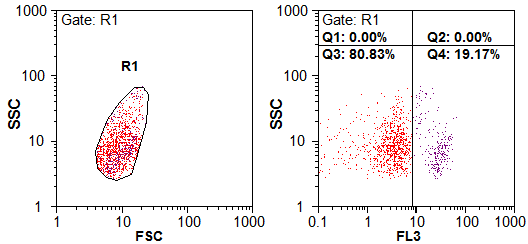

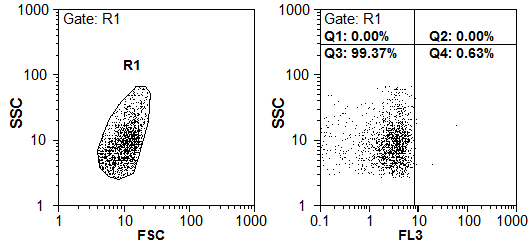

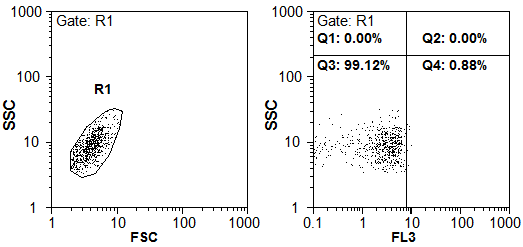

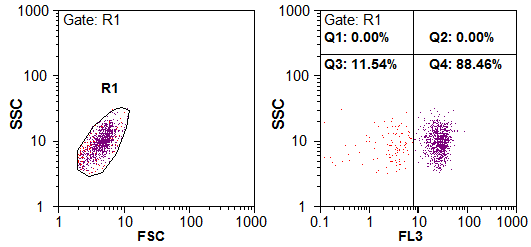


**i. Unlysed ii. Lysed**

Unstained Unstained

PerCP Mouse Anti-Human CD45 stained PerCP Mouse Anti-Human CD45 stained


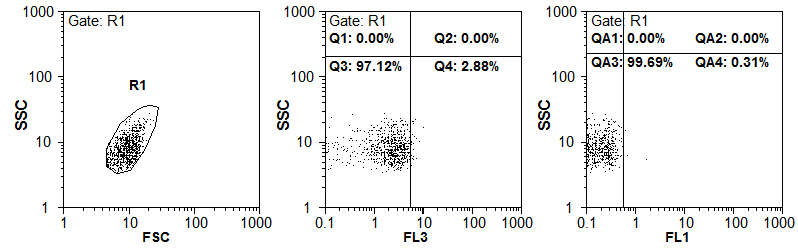

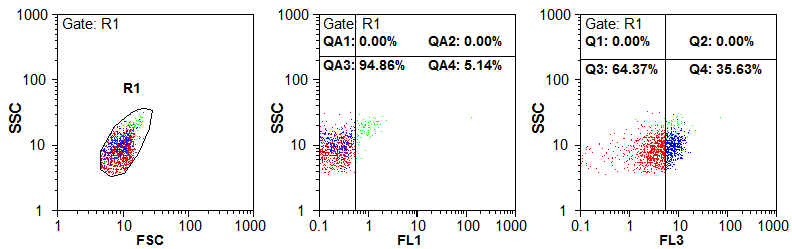

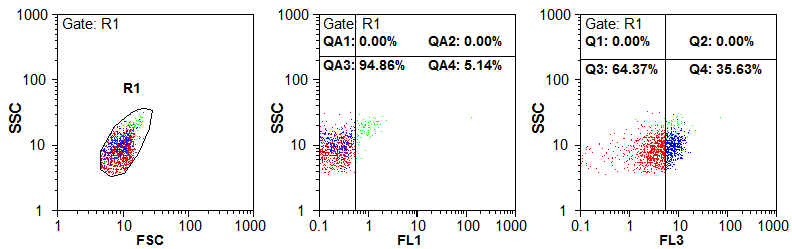

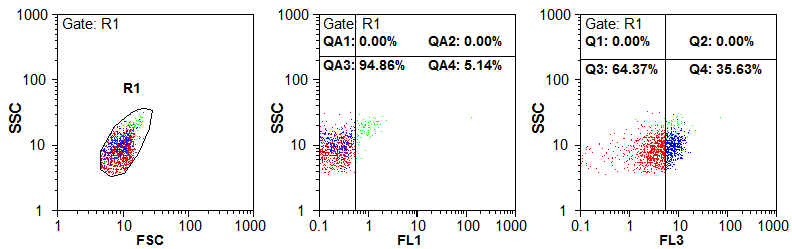


Unstained

PerCP Mouse Anti-Human CD45 and FITC Mouse Anti-Human CD14 stained

**Figure S2. Short term intra-individual stability and day-day precision of superoxide levels, mitochondrial mass and mitochondrial membrane potential.** A. PBMCs, B. Lymphocytes and C. Monocytes. Pearson’s correlation coefficient (r) was used to test the strength of association between day 1 and day 7 intervals and thus *in vivo* stability and the mean coefficient of variation (CV, %) was used to assess day-day assay precision. (n=6).


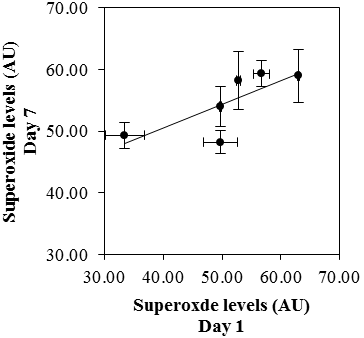

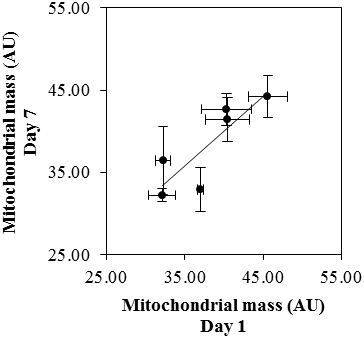

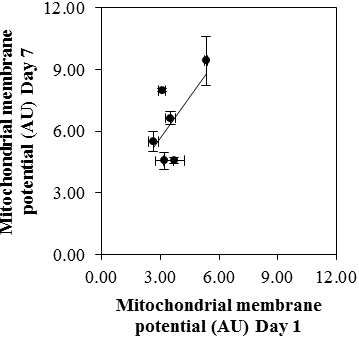

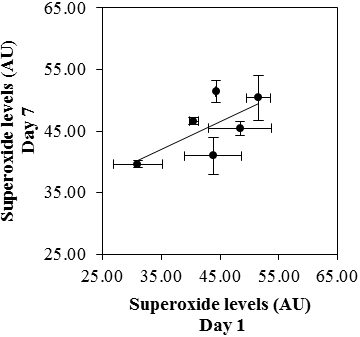

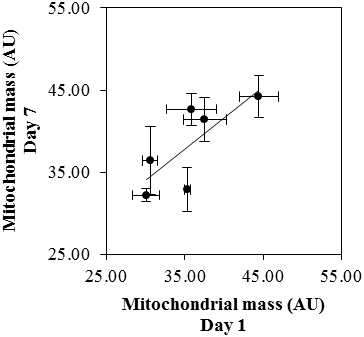

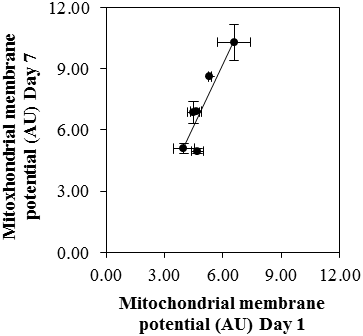

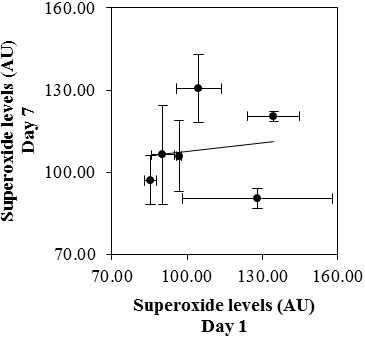

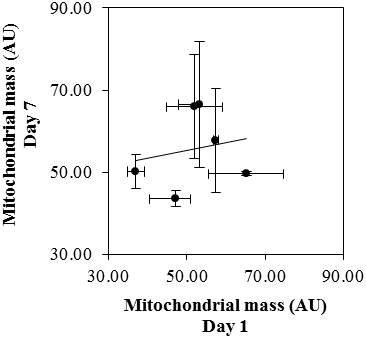

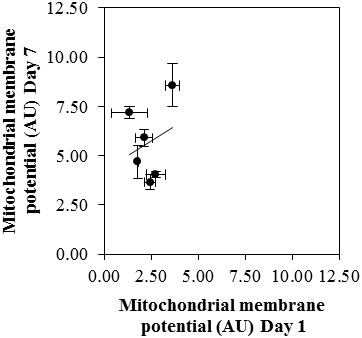


**A.**

**B.**

**C.**

**r = 0.76**

**Mean CV = 8.31%**

**r = 0.85**

**Mean CV = 4.17%**

**r = 0.64**

**Mean CV = 39.02%**

**r = 0.76**

**Mean CV = 5.34%**

**r = 0.90**

**Mean CV = 23.92%**

**r = 0.19**

**Mean CV = 13.19%**

**r = 0.25**

**Mean CV = 56.97%**

**r = 0.14**

**Mean CV = 12.42%**

**r = 0.66**

**Mean CV = 8.09%**

**Figure S3. Scatter plots of agreements between superoxide levels, mitochondrial mass and mitochondrial membrane potential.**


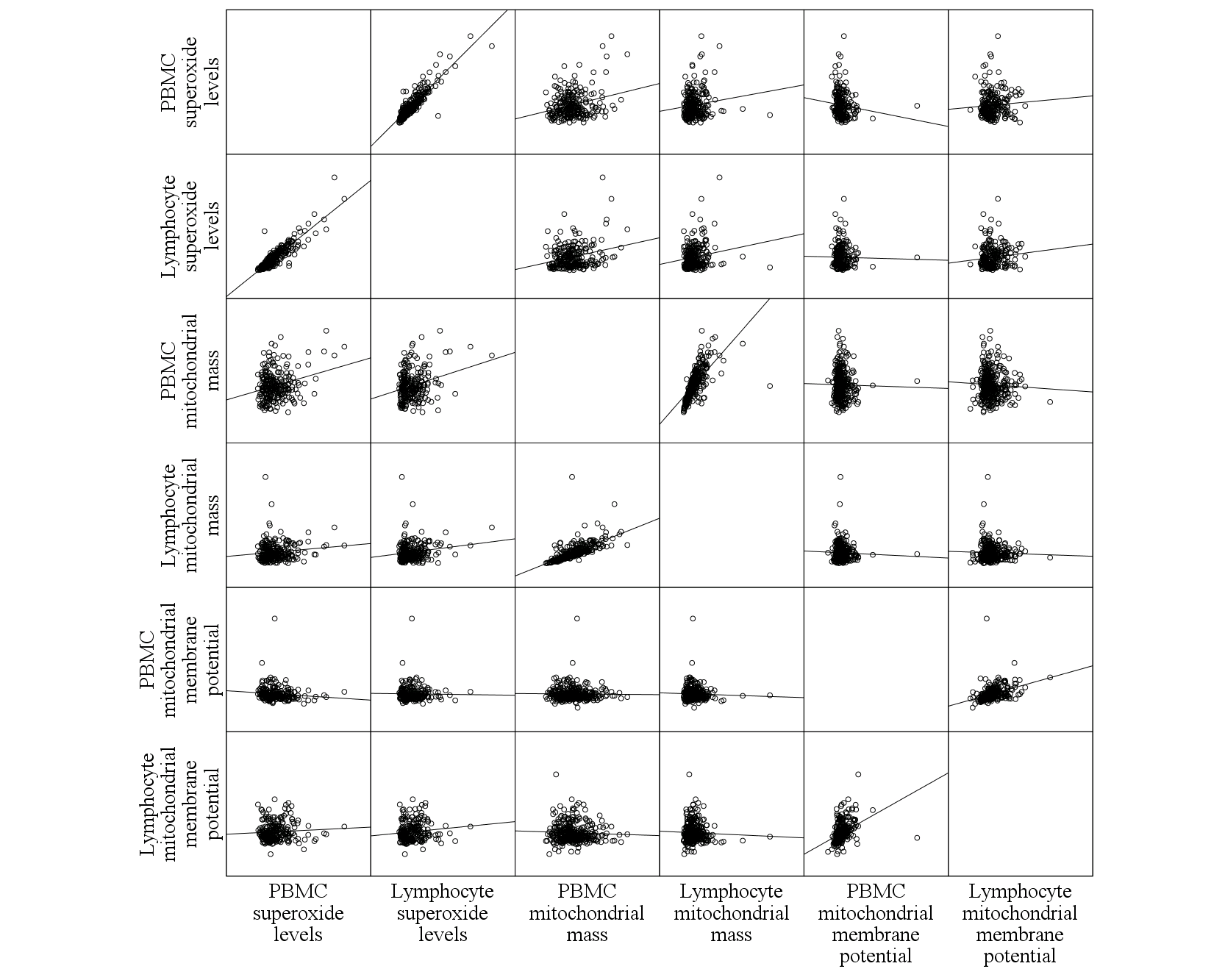


**
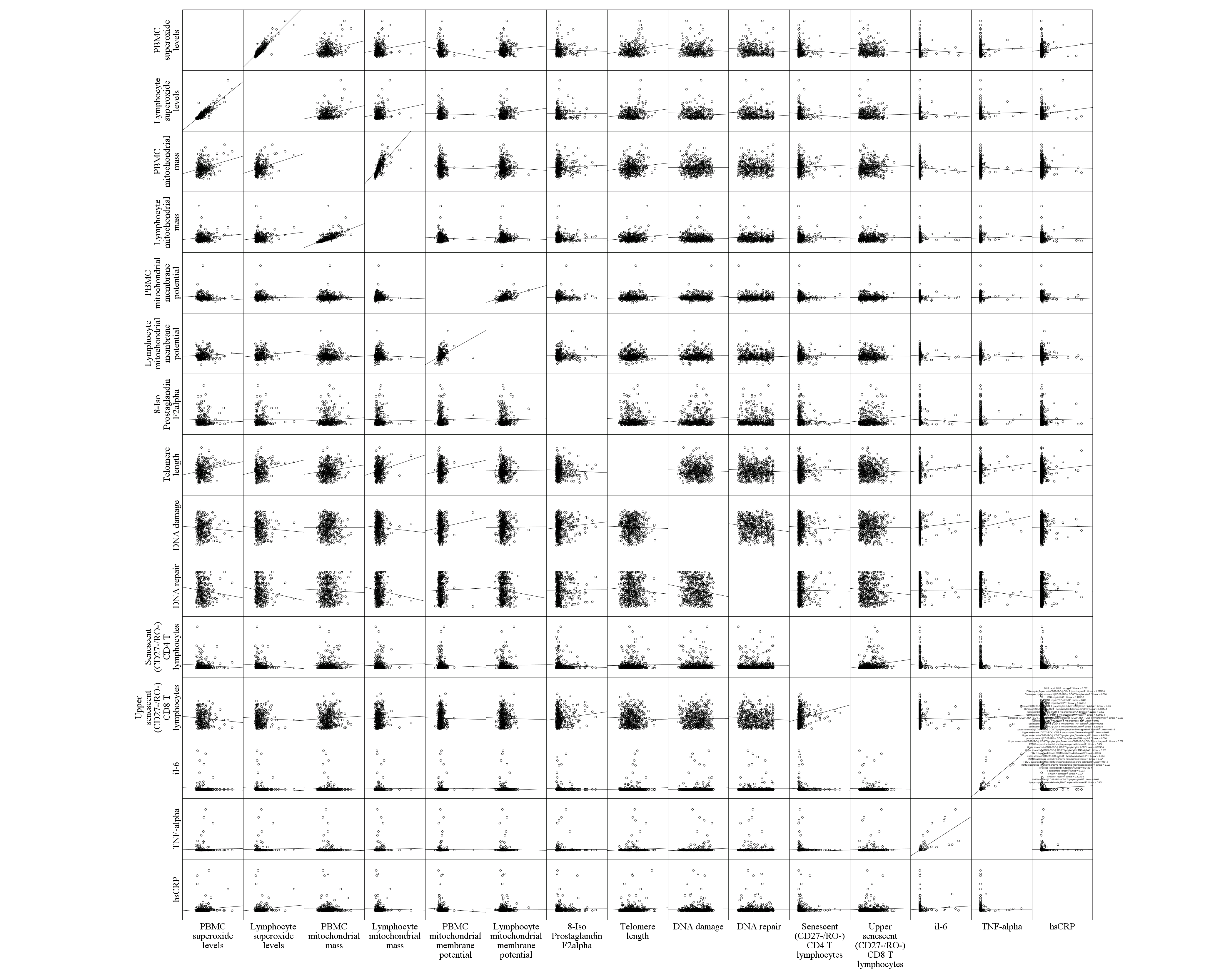
Figure S4. Scatter plots of superoxide levels, mitochondrial mass and mitochondrial membrane potential in relation to other potential markers of oxidative stress-induced cellular senescence.**

**Figure S5. Scatter plots of superoxide levels, mitochondrial mass and mitochondrial membrane potential in relation to other potential markers of oxidative stress-induced cellular senescence after removal of extreme outliers.**


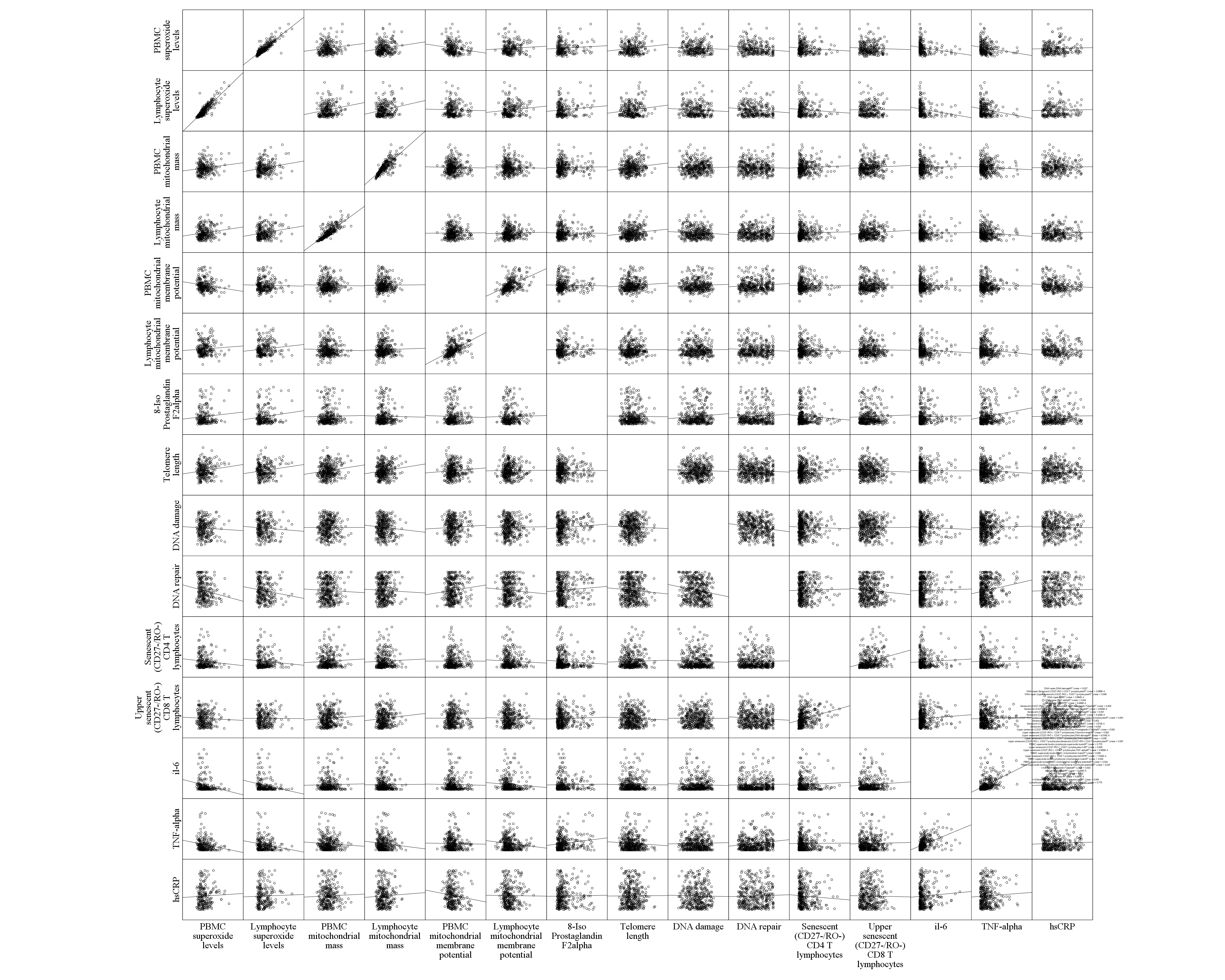


**
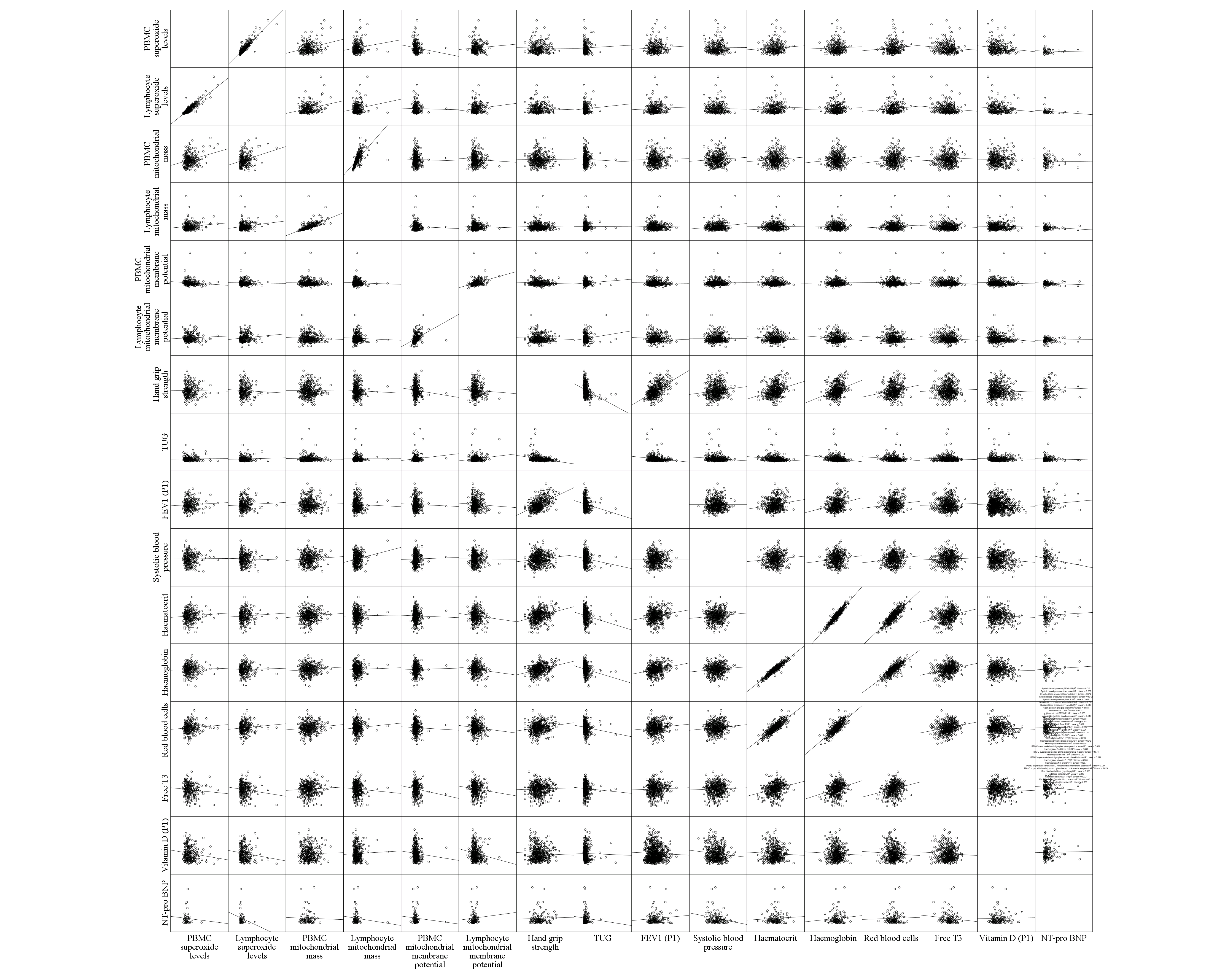
Figure S6.** **Scatter plots of superoxide levels, mitochondrial mass and mitochondrial membrane potential in relation to informative BoA.** (^P1^: baseline (phase 1) data)

**Figure S7.** **Scatter plots of superoxide levels, mitochondrial mass and mitochondrial membrane potential in relation to informative BoA after removal of extreme outliers.** (^P1^: baseline (phase 1) data)


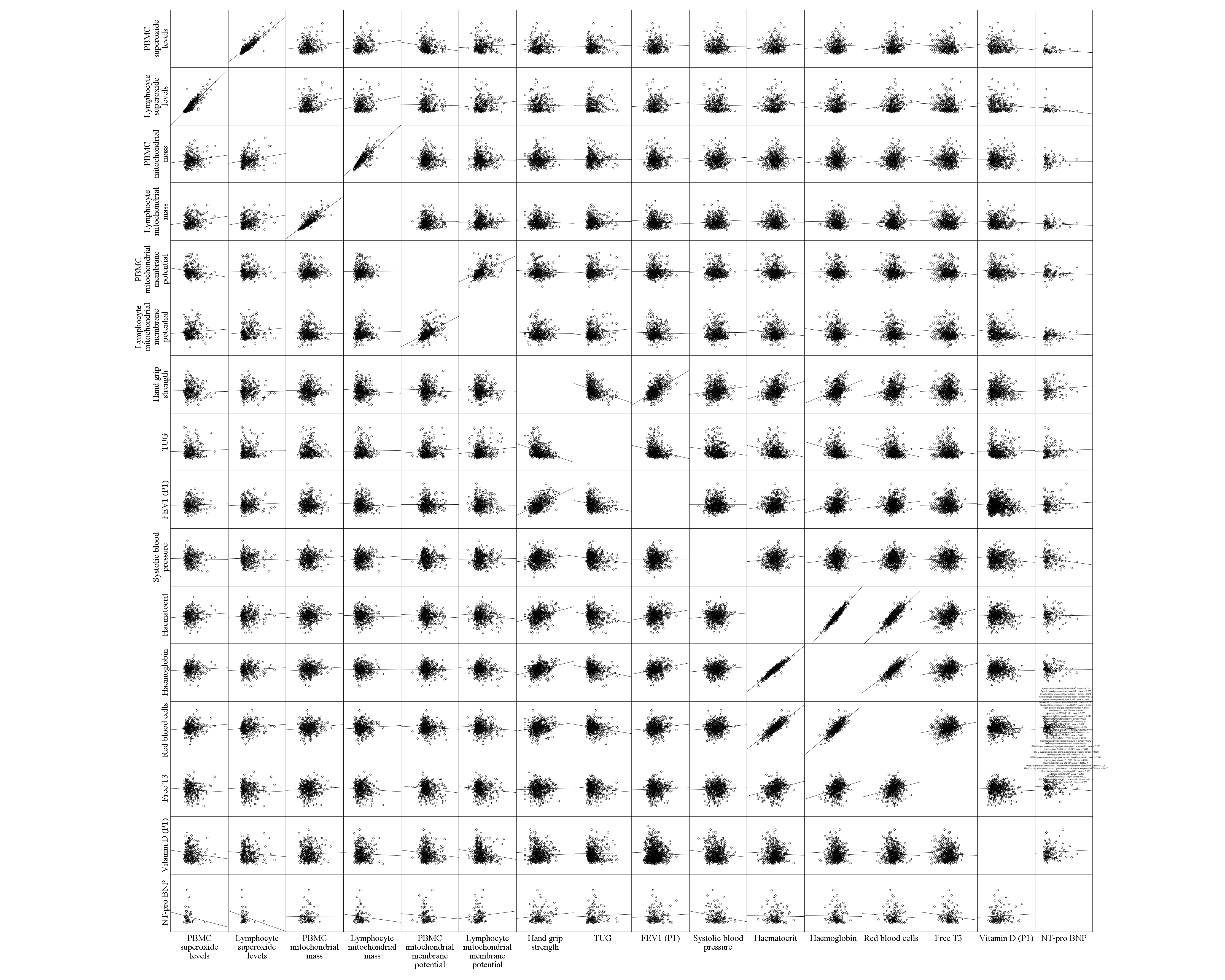


**Table S1. Stability of superoxide levels, mitochondrial mass and mitochondrial membrane potential in PBMCs during various experimental handling.** A. Short-term storage of PBMCs at 37^o^C prior analysis, B. Long-term storage of PBMCs at 37^o^C prior analysis, C. Frozen storage of PBMCs at -70^o^C prior analysis, D. Frozen storage of PBMCs at -196^o^C prior analysis, E. Mechanical stress of PBMCs prior analysis, F. Red blood cell lysis of PBMCs prior analysis, G. Freeze-thaw cycles of stains prior analysis, H. Light exposure of stained PBMCs, I. Incubation temperature of stained PBMCs. Student's t-Test was used to test for the difference between the experimental variable and control (n=3). Where more than one variable was tested, the single experimental variable (^2^ or ^3^) was compared to the control (^1^). (SD: standard deviation).

|  | **Superoxide levels (AU)** | **Mitochondrial mass (AU)** | **Mitochondrial membrane potential (AU)** |
| --- | --- | --- | --- |
|  | **Mean ± SD** | **Mean ± SD** | **Mean ± SD** |
| **A.** 0h (Control) | 39.16 ± 5.53 | 28.53 ± 3.78 | 4.71 ± 0.58 |
| 2h | 32.42 ± 7.42 | 26.06 ± 2.45 | 4.52 ± 0.90 |
| p | 0.28 | 0.40 | 0.77 |
| **B.** 0h (Control) | **28.01 ± 2.54** | 50.83 ± 0.46 | 2.17 ± 0.17 |
| 24h | **80.91 ± 2.02** | 44.04 ± 4.50 | 2.26 ± 0.15 |
| p | **0.00** | 0.06 | 0.54 |
| **C.** Fresh (Control) | **41.03 ± 14.45** | 42.62 ± 4.77 | **5.52 ± 0.98** |
| Frozen | **194.98 ± 15.21** | 46.00 ± 7.99 | **3.28 ± 0.50** |
| p | **0.00** | 0.56 | **0.02** |
| **D.** Fresh (Control) | **53.85 ± 4.56** | **41.47 ± 8.04** | **4.83 ± 0.30** |
| Frozen | **85.9 ± 13.82** | **101.63 ± 8.21** | **2.94 ± 0.27** |
| p | **0.00** | **0.00** | **0.00** |
| **E.** 1000rpm (Control) | 50.69 ± 12.84^1^ | 39.72 ± 2.55^1^ | 3.13 ± 1.59^1^ |
| 2000rpm | 49.63 ± 17.31^2^ | 41.92 ± 2.73^2^ | 3.49 ± 1.23^2^ |
| 3000rpm | 53.83 ± 19.79^3^ | 37.69 ± 0.89^3^ | 2.97 ± 1.16^3^ |
| p | 0.94^1v2^ | 0.37^1v2^ | 0.77^1v2^ |
|  | 0.83^1v3^ | 0.26^1v3^ | 0.90^1v3^ |
| **F.** No lysis (Control) | **31.57 ± 1.68** | 40.05 ± 8.17 | **2.75 ± 0.40** |
| Lysis | **126.31 ± 21.33** | 48.90 ± 5.48 | **1.56 ± 0.06** |
| p | **0.01** | 0.19 | **0.01** |
| **G.** 0 cycles (Control) | 194.98 ± 15.21^1^ | 46.00 ± 7.99^1^ | 3.28 ± 0.50^1^ |
| 5 cycles | 195.65 ± 38.37^2^ | 48.22 ± 4.47^2^ | 3.31 ± 0.54^2^ |
| 10 cycles | 211.94 ± 44.08^3^ | 44.95 ± 6.45^3^ | 3.36 ± 0.60^3^ |
| p | 0.98^1v2^ | 0.71^1v2^ | 0.94^1v2^ |
|  | 0.56^1v3^ | 0.82^1v3^ | 0.86^1v3^ |
| **H.** Unexposed (Control) | 25.70 ± 5.05 | 60.12 ± 14.22 | 8.59 ± 0.62 |
| Exposed | 25.40 ± 4.34 | 52.26 ± 3.59 | 6.41 ± 1.65 |
| p | 0.94 | 0.41 | 0.10 |
| **I.** 37oC (Control) | **55.27 ± 11.18^1^** | **37.81 ± 12.25^1^** | **2.32 ± 0.20^1^** |
| 20^o^C | 34.82 ± 5.54^2^ | 21.37 ± 6.11^2^ | **5.82 ± 0.85^2^** |
| 4^o^C | **25.82 ± 6.33^3^** | **12.23 ± 1.57^3^** | 3.47 ± 1.02^3^ |
| p | 0.05^1v2^ | 0.11^1v2^ | **0.001v2** |
|  | **0.02^1v3^** | **0.02^1v3^** | 0.13^1v3^ |

**Table S2. Agreements between superoxide levels, mitochondrial mass and mitochondrial membrane potential.** (r: Spearman’s correlation coefficient, p: probability, n: number of participants)

|  |  | **Superoxide levels** | | **Mitochondrial mass** | | **Mitochondrial membrane potential** | |
| --- | --- | --- | --- | --- | --- | --- | --- |
|  |  | *PBMCs* | *Lymphocytes* | *PBMCs* | *Lymphocytes* | *PBMCs* | *Lymphocytes* |
| **Superoxide levels** |  |  |  |  |  |  |  |
| *PBMCs* | r | ^_^ | ^_^ | ^_^ | ^_^ | ^_^ | ^_^ |
|  | p | ^_^ | ^_^ | ^_^ | ^_^ | ^_^ | ^_^ |
|  | n | ^_^ | ^_^ | ^_^ | ^_^ | ^_^ | ^_^ |
| *Lymphocytes* | r | **0.88** | ^_^ | ^_^ | ^_^ | ^_^ | ^_^ |
|  | p | **0.00** | ^_^ | ^_^ | ^_^ | ^_^ | ^_^ |
|  | n | **248** | ^_^ | ^_^ | ^_^ | ^_^ | ^_^ |
| **Mitochondrial mass** |  |  |  |  |  |  |  |
| *PBMCs* | r | **0.13** | **0.16** | ^_^ | ^_^ | ^_^ | ^_^ |
|  | p | **0.04** | **0.01** | ^_^ | ^_^ | ^_^ | ^_^ |
|  | n | **243** | **243** | ^_^ | ^_^ | ^_^ | ^_^ |
| *Lymphocytes* | r | **0.13** | **0.17** | **0.84** | ^_^ | ^_^ | ^_^ |
|  | p | **0.04** | **0.01** | **0.00** | ^_^ | ^_^ | ^_^ |
|  | n | **243** | **243** | **341** | ^_^ | ^_^ | ^_^ |
| **Mitochondrial membrane potential** |  |  |  |  |  |  |  |
| *PBMCs* | r | **-0.18** | -0.03 | 0.00 | 0.04 | ^_^ | ^_^ |
|  | p | **0.01** | 0.62 | 0.96 | 0.52 | ^_^ | ^_^ |
|  | n | **239** | 239 | 332 | 332 | ^_^ | ^_^ |
| *Lymphocytes* | r | 0.09 | **0.14** | -0.04 | 0.03 | **0.55** | ^_^ |
|  | p | 0.18 | **0.04** | 0.44 | 0.58 | **0.00** | ^_^ |
|  | n | 239 | **239** | 332 | 332 | **347** | ^_^ |

**Table S3. Agreements between superoxide levels, mitochondrial mass and mitochondrial membrane potential after removal of extreme outliers.** (r: Spearman’s correlation coefficient, p: probability, n: number of participants)

|  |  | **Superoxide levels** | | **Mitochondrial mass** | | **Mitochondrial membrane potential** | |
| --- | --- | --- | --- | --- | --- | --- | --- |
|  |  | *PBMCs* | *Lymphocytes* | *PBMCs* | *Lymphocytes* | *PBMCs* | *Lymphocytes* |
| **Superoxide levels** |  |  |  |  |  |  |  |
| *PBMCs* | r | ^_^ | ^_^ | ^_^ | ^_^ | ^_^ | ^_^ |
|  | p | ^_^ | ^_^ | ^_^ | ^_^ | ^_^ | ^_^ |
|  | n | ^_^ | ^_^ | ^_^ | ^_^ | ^_^ | ^_^ |
| *Lymphocytes* | r | **0.87** | ^_^ | ^_^ | ^_^ | ^_^ | ^_^ |
|  | p | **0.00** | ^_^ | ^_^ | ^_^ | ^_^ | ^_^ |
|  | n | **245** | ^_^ | ^_^ | ^_^ | ^_^ | ^_^ |
| **Mitochondrial mass** |  |  |  |  |  |  |  |
| *PBMCs* | r | 0.10 | **0.14** | ^_^ | ^_^ | ^_^ | ^_^ |
|  | p | 0.13 | **0.03** | ^_^ | ^_^ | ^_^ | ^_^ |
|  | n | 240 | **241** | ^_^ | ^_^ | ^_^ | ^_^ |
| *Lymphocytes* | r | **0.13** | **0.17** | **0.83** | ^_^ | ^_^ | ^_^ |
|  | p | **0.05** | **0.01** | **0.00** | ^_^ | ^_^ | ^_^ |
|  | n | **236** | **237** | **337** | ^_^ | ^_^ | ^_^ |
| **Mitochondrial membrane potential** |  |  |  |  |  |  |  |
| *PBMCs* | r | **-0.18** | -0.04 | 0.01 | 0.06 | ^_^ | ^_^ |
|  | p | **0.01** | 0.53 | 0.88 | 0.27 | ^_^ | ^_^ |
|  | n | **234** | 235 | 328 | 324 | ^_^ | ^_^ |
| *Lymphocytes* | r | 0.08 | **0.13** | -0.04 | 0.06 | **0.54** | ^_^ |
|  | p | 0.21 | **0.05** | 0.52 | 0.29 | **0.00** | ^_^ |
|  | n | 237 | **238** | 331 | 327 | **343** | ^_^ |

**Table S4: Superoxide levels, mitochondrial mass and mitochondrial membrane potential in relation to other potential markers of oxidative stress-induced cellular senescence after removal of extreme outliers.** (r: Spearman’s correlation coefficient, p: probability, n: number of participants)

|  |  | **Superoxide levels** | | **Mitochondrial mass** | | **Mitochondrial membrane potential** | |
| --- | --- | --- | --- | --- | --- | --- | --- |
|  |  | *PBMCs* | *Lymphocytes* | *PBMCs* | *Lymphocytes* | *PBMCs* | *Lymphocytes* |
| **8-iso Prostaglandin F_2α_** | r | -0.01 | -0.01 | 0.05 | 0.03 | 0.02 | 0.05 |
|  | p | 0.87 | 0.88 | 0.42 | 0.56 | 0.71 | 0.43 |
|  | n | 201 | 202 | 286 | 282 | 284 | 287 |
| **Telomere length** | r | 0.08 | 0.06 | **0.11** | **0.12** | **0.12** | 0.04 |
|  | p | 0.19 | 0.36 | **0.04** | **0.03** | **0.03** | 0.41 |
|  | n | 245 | 246 | **340** | **336** | **342** | 345 |
| **DNA damage** | r | -0.05 | -0.09 | 0.05 | -0.04 | 0.03 | 0.03 |
|  | p | 0.39 | 0.17 | 0.38 | 0.51 | 0.63 | 0.62 |
|  | n | 245 | 246 | 341 | 337 | 343 | 346 |
| **DNA repair** | r | **-0.17** | **-0.15** | -0.06 | -0.05 | 0.07 | -0.02 |
|  | p | **0.01** | **0.02** | 0.26 | 0.33 | 0.19 | 0.65 |
|  | n | **245** | **246** | 341 | 337 | 343 | 346 |
| **CD27-/RO- CD4 T lymphocytes** | r | -0.09 | -0.04 | 0.07 | 0.09 | 0.06 | 0.00 |
|  | p | 0.19 | 0.53 | 0.23 | 0.12 | 0.32 | 0.93 |
|  | n | 228 | 229 | 315 | 312 | 317 | 320 |
| **CD27-/RO- CD8 T lymphocytes** | r | -0.07 | -0.03 | 0.09 | 0.10 | 0.03 | -0.02 |
|  | p | 0.27 | 0.70 | 0.12 | 0.09 | 0.58 | 0.75 |
|  | n | 234 | 235 | 317 | 313 | 321 | 323 |
| **Il-6** | r | **-0.30** | **-0.30** | 0.02 | -0.06 | -0.07 | **-0.18** |
|  | p | **0.00** | **0.00** | 0.73 | 0.29 | 0.25 | **0.00** |
|  | n | **207** | **208** | 296 | 292 | 299 | **302** |
| **TNF**-α | r | **-0.20** | **-0.20** | -0.04 | -0.10 | -0.04 | **-0.12** |
|  | p | **0.00** | **0.00** | 0.54 | 0.07 | 0.53 | **0.03** |
|  | n | **215** | **216** | 310 | 306 | 312 | **315** |
| **hsCRP** | r | 0.03 | 0.06 | 0.07 | 0.03 | **-0.15** | -0.09 |
|  | p | 0.65 | 0.38 | 0.27 | 0.63 | **0.01** | 0.15 |
|  | n | 201 | 202 | 274 | 270 | **277** | 280 |

**Table S5: Superoxide levels, mitochondrial mass and mitochondrial membrane potential in relation to informative BoA after removal of extreme outliers.** (r: Spearman’s correlation coefficient, p: probability, n: number of participants, ^P1^: baseline (phase 1) data)

|  |  | **Superoxide levels** | | **Mitochondrial mass** | | **Mitochondrial membrane potential** | |
| --- | --- | --- | --- | --- | --- | --- | --- |
|  |  | *PBMCs* | *Lymphocytes* | *PBMCs* | *Lymphocytes* | *PBMCs* | *Lymphocytes* |
| **Hand grip strength** | r | 0.01 | 0.00 | -0.02 | -0.05 | -0.04 | 0.00 |
|  | p | 0.91 | 0.99 | 0.71 | 0.34 | 0.42 | 0.98 |
|  | n | 240 | 241 | 336 | 332 | 338 | 341 |
| **TUG** | r | 0.00 | -0.02 | 0.04 | 0.04 | 0.06 | 0.08 |
|  | p | 0.95 | 0.80 | 0.49 | 0.44 | 0.32 | 0.17 |
|  | n | 213 | 214 | 299 | 295 | 304 | 306 |
| **FEV1 ^P1^** | r | 0.05 | 0.06 | 0.02 | -0.04 | 0.02 | 0.00 |
|  | p | 0.41 | 0.35 | 0.77 | 0.45 | 0.72 | 0.95 |
|  | n | 241 | 242 | 335 | 331 | 337 | 340 |
| **Systolic blood pressure** | r | 0.01 | -0.02 | 0.07 | 0.08 | 0.01 | 0.01 |
|  | p | 0.90 | 0.78 | 0.22 | 0.14 | 0.82 | 0.78 |
|  | n | 242 | 243 | 338 | 334 | 339 | 342 |
| **Haematocrit** | r | 0.06 | 0.07 | 0.05 | -0.04 | -0.04 | -0.05 |
|  | p | 0.36 | 0.24 | 0.35 | 0.42 | 0.50 | 0.36 |
|  | n | 243 | 244 | 338 | 334 | 340 | 343 |
| **Haemoglobin** | r | 0.06 | 0.08 | 0.08 | -0.02 | 0.01 | -0.06 |
|  | p | 0.39 | 0.20 | 0.16 | 0.66 | 0.89 | 0.29 |
|  | n | 243 | 244 | 338 | 334 | 340 | 343 |
| **Red blood cells** | r | 0.09 | 0.12 | 0.10 | 0.04 | -0.03 | -0.08 |
|  | p | 0.17 | 0.06 | 0.08 | 0.48 | 0.62 | 0.13 |
|  | n | 243 | 244 | 338 | 334 | 340 | 343 |
| **Free T3** | r | -0.08 | -0.10 | 0.03 | -0.03 | -0.07 | **-0.12** |
|  | p | 0.20 | 0.11 | 0.61 | 0.62 | 0.19 | **0.03** |
|  | n | 243 | 244 | 338 | 334 | 340 | **343** |
| **Vitamin D ^P1^** | r | -0.11 | -0.11 | -0.01 | -0.07 | -0.10 | **-0.18** |
|  | p | 0.08 | 0.08 | 0.89 | 0.18 | 0.06 | **0.00** |
|  | n | 237 | 238 | 331 | 327 | 334 | **337** |
| **NT-pro BNP** | r | -0.16 | -0.18 | -0.09 | -0.13 | -0.09 | 0.12 |
|  | p | 0.31 | 0.25 | 0.44 | 0.24 | 0.4 | 0.26 |
|  | n | 46 | 46 | 85 | 85 | 95 | 95 |

**Table S6: Association between superoxide levels, mitochondrial mass and mitochondrial membrane potential and age-related outcomes after removal of extreme outliers.** (r: Spearman’s correlation coefficient, p: probability, n: number of participants)

|  |  | **Superoxide levels** | | **Mitochondrial mass** | | **Mitochondrial membrane potential** | |
| --- | --- | --- | --- | --- | --- | --- | --- |
|  |  | *PBMCs* | *Lymphocytes* | *PBMCs* | *Lymphocytes* | *PBMCs* | *Lymphocytes* |
| **Disability score** | r | 0.01 | 0.01 | -0.03 | 0.01 | 0.01 | 0.06 |
|  | p | 0.82 | 0.90 | 0.60 | 0.85 | 0.89 | 0.27 |
|  | n | 244 | 245 | 339 | 335 | 341 | 344 |
| **SMMSE score** | r | 0.07 | 0.08 | 0.08 | 0.03 | 0.05 | -0.09 |
|  | p | 0.30 | 0.24 | 0.13 | 0.55 | 0.40 | 0.11 |
|  | n | 244 | 245 | 339 | 335 | 341 | 344 |
| **Disease count** | r | 0.1 | 0.08 | 0.04 | 0.06 | -0.04 | -0.02 |
|  | p | 0.11 | 0.19 | 0.46 | 0.24 | 0.48 | 0.77 |
|  | n | 236 | 237 | 328 | 324 | 330 | 333 |

**Supplementary Methods**

***Anthropometry, blood pressure and physical function***

*Data on weight, body fat percentage, body fat mass, fat free mass and total body water* were obtained using a Tanita electronic body composition analyser (Tanita Europe B.V. Middlesex, UK). In view of the well recognized difficulties in measuring height of very old people, height was estimated from demi-span. *Right arm demi-span* was measured to the nearest 0.1cm; final data are the average of two measurements. The formulae for calculating height are: [1.35 x demi-span + 60.1] for women and [1.40 x demi-span + 57.8] for men. *Hip and waist circumferences* were measured to the nearest 0.1cm.

*Diastolic and systolic blood pressures* were measured using a digital blood pressure monitor- Omron HEM 705-IT (Omron Healthcare UK Ltd., Milton Keynes, UK). Three measurements were taken with 2 minute intervals in-between; the average of the second and third measurements was used.

*Hand grip strength* was measured twice in each hand using a electronic dynamometer (Takei Physical Fitness Test, Chasmors Ltd, UK). Participants with localised pathology affecting the upper limb were not excluded. The device was adjusted to the participant’s hand size and recordings made in the sitting position with the forearm resting, the upper arm adducted into the body and the elbow bent at 90˚. The mean of the highest readings from each hand was used in the analysis.

*The Timed up-and-go (TUG) test time:* Using a stop watch, the time in seconds taken to rise from a standard chair, walk 3 metres, turn, walk back and sit down was recorded (1). A standard chair (46cm seat height, 64cm arm height) was taken to the participant’s home.

*Respiratory function* was measured using a MicroLab Spirometer with Spida 5 software (Micro Medical Ltd., Rochester, UK). The aim was to obtain three technically satisfactory 'blows'; measurements were repeated until this was achieved or the maximum effort for the participant was reached. Blows were assessed for technical adequacy using in-built Spida algorithms with additional visual inspection of flow-volume loops where necessary. The highest value from any good quality blow for Forced Expiratory Volume in one second (FEV1), Forced Vital Capacity (FVC) and Peak Expiratory Flow Rate (PEFR) was used in the analysis.

***Blood-based candidate biomarkers***

*Haematology and biochemistry:* full blood count; electrolytes, urea, creatinine and urate; liver panel (total protein, bilirubin and alanine transaminase (ALT)); bone panel (albumin, calcium and albumin‑adjusted calcium, phosphate and alkaline phosphatase (ALP)); glucose and glycosylated haemoglobin (HbA1c, Tosoh Eurogenetics automated HLC-723G7 HPLC analyser); lipid profile (cholesterol, triglycerides, high and low density lipoproteins, apolipoproteins (A1 and B)); serum cortisol; thyroid function (free Triiodothyronine (T3), free thyroxine (T4), thyroid stimulating hormone (TSH) and thyroid peroxidase antibody (ATPO) (ADVIA Centaur chemiluminescence immunoassay)); high-sensitivity C-reactive protein (hsCRP, Dade Behring CardioPhase hsCRP immunoassay); rheumatoid factor; and N-terminal pro b-type natriuretic peptide (NT-proBNP).

*Nutritional markers:* serum ferritin by immunoradiometric assay (Ferritin Mab; ICN Pharmaceuticals); plasma total homocysteine (Abbott IMx immunoassay); plasma vitamin B2 by erythrocyte glutathione reductase activation coefficient (EGRAC) on a Cobas Bio centrifugal analyzer; plasma vitamin B6, both pyridoxal phosphate (PLP, active form) and pyridoxic acid (PA, inactive catabolite) by reversed-phase HPLC‑fluorescence with chlorite postcolumn derivatization (2); red cell folate and vitamin B12 by chemiluminescence (Microparticle Immunoassay on Abbott ARCHITECT analyser); and serum vitamin D (Diasorin 25-hydroxy-vitamin D assay).

*Inflammatory response:* LiHep blood samples were stimulated for cytokine production with Invivogen Ultrapure LPS (Autogenbioclear, Nottingham, UK) and after 24h incubation at 37˚C serum supernatants were collected; IL-6 and TNF-alpha were assessed by electrochemiluminescence on a 96-well Multi-SPOT Meso Scale Discovery assay with a SECTOR Imager 6000 (Meso Scale Diagnostics, LLC. Gaithersburg, MD, USA).

*Lymphocyte subpopulations* were determined to measure clonality of these cells types for interpretion of other measures such as telomere length; blood samples were analysed by 4-colour flow-cytometry (Becton Dickinson FACScan Flow Cytometer) for: B Cells (CD19+) Memory (CD27+) and Naive B (CD27-); CD8 T cells (CD3+/CD8+) Memory (CD45RO+/CD27-) and Naive (CD45RO-/CD27+); CD4 T cells (CD4+) Memory (CD45RO+/CD27-) and Naive (CD45RO-/CD27+). All fluorescence-labelled antibodies were obtained from BD Biosciences (Oxford, UK).

*Telomere length* was measured as abundance of telomeric template versus a single gene (GAPDH) by quantitative real-time PCR (3). The intra-assay coefficient of variation was 2.7% while the inter-assay coefficient of variation was 5.1%. Four internal control DNA samples were run within each plate to correct for plate–to-plate variation. Measurements were performed in quadruplicate. All PCRs were carried out on an Applied Biosystems 7900HT Fast Real Time PCR machine with 384-well plate capacity.

*DNA Damage and Repair*: DNA Radiosensitivity (damage) and DNA repair capacity of PBMCs were assessed by automated fluorimetric alkaline DNA unwinding (FADU) analysis as described (4) with the following modifications: PBMC recovered from LiHep blood samples were kept in culture with RPMI 1640 medium plus 10% FBS, 1% Pen/Strep and 2% Glutamine at 37˚C, 5%CO_2_ overnight. Three 50 μl aliquots of 5x10^5^ cells were prepared per participant: control cells (C), damaged cells (D) which were treated with 5 Gy of gamma-irradiation (a dose estimated to halve the integrity of the DNA through single strand breaks), and repair cells (R) which after 5Gy of gamma-irradiation are kept 1h at 37˚C to allow for the damage to be repaired. These cell suspensions were kept at all times on ice. Samples were then treated with 10 mM sodium phosphate, 0.25 M meso-inositol, 1mM MgCl_2_, and pH 7.2 and applied onto 96-well black microplates, with 6 repeats for each of the conditions described above. By means of an epMotion liquid handling robot with thermal modules (Eppendorf UK Ltd, Histon, UK) cells were sequentially treated as follows: 12min at 4˚C with lysis buffer (9 M Urea, 10 mM NaOH, 25 mM CDTA; 0.1 % SDS), 15 min at 4˚C plus 90 min incubation at 30˚C with alkaline buffer (40% lysis buffer in 0.2 M NaOH), 10min at RT with neutralising buffer (1 M Glucose, 15 mM beta-mercaptoethanol) and 7 min at RT in the dark with 1xSYBR Green (diluted in 48mM NaOH). The plates were then measured for fluorescence (excitaton 485nm, emission 535nm) on a TECAN SPECTRAFluor Plus fluorimeter (TECAN UK, Reading, UK). Each plate included an internal control of Sheared Salmon Sperm DNA, 60ng/μl. DNA Damage (D) was estimated as % of fluorescence signal intensity in samples C that is lost in samples D, while DNA Repair (R) is estimated as % of that loss that has been recovered on samples R. The coefficient of variation for these measurements was 10.8%.

**D** = 100 x 1

-

average signal intensity for samples D

average signal intensity for samples C

**R** = 100

-

100 x

B

D

-

average signal intensity for samples R

average signal intensity for samples C

DNA Damage remaining after 1h Repair (**B**) = 100 x 1


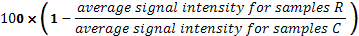


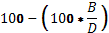


*Plasma Isoprostanes by LC/MS/MS:* Isoprostanes iPF2α-III and iPF2α-VI were extracted from EDTA-plasma samples (300μl) and measured by LC-MSMS as described (5) with modifications as follows: Isoprostanes, iPF2α-III and iPF2α-VI, and the deuterated analogue iPF2α-III–D4 as internal standard, were obtained from Cayman Chemical (Ann Arbor, MI, USA) and diluted in LC-MS grade methanol. Calibration curve standards and EDTA-plasma samples (300μl) were spiked with 2ng of iPF2α-III–D4, treated with 500μL of aqueous 1M KOH and incubated at 37˚C for 14h. After incubation, pH of the samples was adjusted to pH 3 with 2% orthophosphoric acid and centrifuged at 13000 rpm. Samples were then applied for purification onto 96 well Bond Elute Plexa solid phase extraction (SPE) cartridges (100mg, Varian, Harbor City, CA, USA) preconditioned with water and methanol. After washing with 5% methanol, the isoprostanes were eluted with methanol. Eluates were evaporated to dryness under a stream of N2 and reconstituted in 100μL of 90:10 water:methanol. A seven point linear calibration curve was established over a range of 0.1-10ng/ml for both iPF2α-III and iPF2α-VI. The seven calibration standards were run both before and after the participants' samples. Every 20 samples, an internal control sample (10%methanol spike with iPF2α-III and iPF2α-VI) was run to monitor intra-assay and inter-assay variability. Quantitation of plasma iPF2α-III and iPF22α-VI was performed using a Quantum Ultra triple quadrupole mass spectrometer coupled to a Surveyor LC system operating with Xcalibur software, version 2.0 (ThermoFisher Scientific, Hemel Hempstead, UK). A Rheodyne 10 port switching valve was used for on-line SPE (2mm x 20mm Strata C18-E 20 µm cartridge, Phenomenex,Macclesfield, UK). Samples were loaded onto the on-line extraction cartridge with 80:20 water:methanol at a flow of 1ml/minute.Liquid chromatographic separation was carried out on a 2.1mm x 50mm x 2.5μm XBridge C18 column (Waters, Manchester, UK), with gradient elution comprising solvent A of 20mM (NH4)2CO3/NH4HCO3 in water and solvent B of 20mM (NH4)2CO3/NH4HCO3 in 95:5 methanol: water, at pH 9.2 and a flow rate of 200μL/min. A solvent gradient of 90% A, 10%B to 40%A and 60%B over 20minutes, followed by 100% B for a further 5 minutes was employed. The analytical runs were performed at 40^o^C. The mass spectrometer was operated using multiple reaction monitoring (MRM) in negative ion mode, with a heated electrospray ionisation source (h-ESI) with argon as collision gas. Precursor, product ions and collision energy were determined after optimising of MS/MS conditions by infusion of 100μg/mL solutions of iPF2α-III, iPF2α-VI and iPF2α-III–D4 in methanol. Both iPF2α-III and iPF2α-VI had a parent ion m/z of 353.2, with iPF22α-III giving a diagnostic product ion m/z of 193.1, while iPF22α-VI gave a diagnostic product ion m/z of 115.1. The internal standard, iPF2α-III–D4, had a parent ion m/z of 357.2, giving a diagnostic product ion m/z of 197.1.

*Plasma Isoprostanes by AutoDELFIA:* LiHep plasma samples were assessed for iPF_2α_-III content by competitive assay on an automated europium (Eu^3+^)-based Dissociation Enhanced Lanthanide Fluorescence Immunoassay (AutoDELFIA automatic immunoassay system, Perkin Elmer, Cambridge, UK). A six-point linear standard calibration curve (0‑100ng/ml, 0.25 ng/ml as lowest sensitivity limit for the assay) and 3 internal controls (50, 2 and 0.5ng/ml) were generated with iPF_2α_-III (Cayman Chemical, Ann Arbor, MI, USA) diluted in PBS +0.5% Ovalbumin. Fifty microliters of sample (plasma, internal controls or standard curve dilutions) were applied onto DELFIA® Yellow Low Fluorescence 96-well plates coated with rabbit anti-mouse antibody. A primary monoclonal antibody (anti‑ iPF_2α_-III antibody, clon 6514:14, Unilever, Cloworth, UK) at a final concentration of 30ng/μl was applied to the samples, as well as 8.375pg/ml of Eu^3+^-labelled ovalbumin- iPF_2α_-III tracer. Following 1h incubation and six washes, enhancement solution was applied and after 5min iPF_2α_-III fluorescence was quantified by means of Multicalc (Perkin-Elmer). The intra-assay coefficient of variation was below 10% while the inter-assay coefficient of variation was 7.4%.

**Supplementary References**

1 Podsiadlo D, Richardson S. The timed up and go - a test of basic functional mobility for frail elderly persons. *J Am Geriatr Soc*.1991;39 142-148.

2 Rybak ME, Pfeiffer CM. Clinical analysis of vitamin B(6): Determination of pyridoxal 5'-phosphate and 4-pyridoxic acid in human serum by reversed-phase high-performance liquid chromatography with chlorite postcolumn derivatization. *Anal Biochem*. 2004;333 (2):336-344.

3 Martin-Ruiz CM, Gussekloo J, van Heemst D, von Zglinicki T, Westendorp RG. Telomere length in white blood cells is not associated with morbidity or mortality in the oldest old: A population-based study. *Aging Cell*. 2005;4 (6):287-290.

4 Moreno-Villanueva M, Pfeiffer R, Sindlinger T, Leake A, Muller M, Kirkwood TB, Burkle A. A modified and automated version of the 'fluorimetric detection of alkaline DNA unwinding' method to quantify formation and repair of DNA strand breaks. *BMC Biotechnol*.2009;9 39.

5 Liang Y, Wei P, Duke RW, Reaven PD, Harman SM, Cutler RG, Heward CB. Quantification of 8-iso-prostaglandin-f(2alpha) and 2,3-dinor-8-iso-prostaglandin-f(2alpha) in human urine using liquid chromatography-tandem mass spectrometry. *Free Radic Biol Med*.2003;34 (4):409-418.
